# Exploring the Antioxidant, Anti-Inflammatory and Skin-Enzyme Inhibitory Activities of Balkan Ethnomedicinal Herbs Through In Vitro and In Vivo Screening

**DOI:** 10.3390/molecules31091524

**Published:** 2026-05-04

**Authors:** Zoi Kardasi, Evanthia Dina, Zora Dajić-Stevanović, Dimitris Ourailoglou, Nektarios Aligiannis, Angeliki P. Kourounakis

**Affiliations:** 1 Department of Medicinal Chemistry, Faculty of Pharmacy, School of Health Sciences, National & Kapodistrian University of Athens, 15771 Athens, Greece; zoikardasi@gmail.com; 2 Department of Pharmacognosy & Chemistry of Natural Products, Faculty of Pharmacy, School of Health Sciences, National & Kapodistrian University of Athens, 15771 Athens, Greece; euantina@pharm.uoa.gr (E.D.); dimioura@pharm.uoa.gr (D.O.); 3 Unity of Dermatopharmacology, Department of Pharmaceutical Technology, Faculty of Pharmacy, School of Health Sciences, National & Kapodistrian University of Athens, 15771 Athens, Greece; 4 Faculty of Agriculture, University of Belgrade, Nemanjina 6, 11080 Belgrade, Serbia; dajic@agrif.bg.ac.rs

**Keywords:** Balkan aromatic and medicinal plants, antioxidant, anti-inflammatory, collagenase inhibition, anti-tyrosinase activity, HPTLC, COX, LOX, DPPH, mouse paw edema

## Abstract

This study aims to evaluate the antioxidant and anti-inflammatory potential of dichloromethanic, methanolic and hydroalcoholic extracts of seventeen different selected Balkan medicinal herbs with ethnopharmacological interest, with the goal of identifying the most bioactive candidates for further investigation of their therapeutic efficacy in human diseases. A total of fifty-four extracts were initially screened; due to the high sample number, only the most active samples were advanced to subsequent assays in order to identify bioactive candidates with potential therapeutic efficacy in human diseases. The methanolic extract of *Cotinus coggygria* showed the highest radical scavenging activity (DPPH: 96.4% inhibition), the hydroalcoholic extract of *Hypericum empetrifolium* exhibited the most potent iron chelation (IC_50_: 5.0 μg/mL) and the methanolic extract of *Sedum sediforme* presented the best anti-inflammatory activity in in vitro assays (LOX IC_50_: 39.4 μg/mL, COX-1 inhibition: 93.1% and COX-2 inhibition: 94.0%). Furthermore, significant inhibition of tyrosinase and collagenase was observed for the methanolic extract of *Cistus creticus* (94.2% tyrosinase inhibition, 86.8% collagenase inhibition) and the methanolic extract of *Cotinus coggygria* (83.1% tyrosinase inhibition, 96.1% collagenase inhibition). In vivo, five promising plant extracts were selected and evaluated for their anti-inflammatory activity using a carrageenan-induced paw edema model in female C57BL/6 mice. The study aimed to assess the in vivo anti-inflammatory potential of these extracts under acute inflammatory conditions. The methanolic extract of Cotinus coggygria proved the most active, significantly reducing paw edema by 34% compared to the non-treated control, indicating a pronounced anti-inflammatory effect and supporting its potential as a source of bioactive compounds with therapeutic relevance. The results of this study indicate that several selected herbal extracts exhibit notable pharmacological activities. Given their antioxidant, anti-inflammatory, and inhibitory properties against enzymes related to skin function, these extracts warrant further in vivo and (pre)clinical investigation for potential use in cosmetic and pharmaceutical products targeting skin disorders associated with inflammation and oxidative stress.

## 1. Introduction

Throughout history, medicinal plants with bioactive compounds have been employed in traditional medicine to treat various health conditions. Empirical evidence gathered over centuries has led to the identification of specific plants with therapeutic effects, particularly for skin diseases, like wounds, hemorrhoids, boils, eczema, excrescences and hair loss. Their antibacterial, anti-inflammatory and antioxidant effects contribute to the healing process of the skin. The use of plant extracts for medicinal purposes strongly suggests that some pharmacologically active components are present in these plant extracts, while they mainly belong to the classes of alkaloids, carotenoids, flavonoids, tannins, terpenoids, saponins, and phenolic compounds [1]. Therefore, the investigation of the pharmacological properties of extracts derived from medicinal plants has gathered significant scientific attention due to their potential therapeutic benefits [2]. Research efforts over the past decades have focused on the discovery of natural products exhibiting anti-inflammatory, immunomodulatory, antioxidant, and chemopreventive properties for the treatment of various diseases [3]. In biological systems, studying the mechanisms by which plant extracts demonstrate their activities is complex and dependent on their overall composition [2].

In traditional Mediterranean and Balkan medicine, numerous native plant species have been employed for the treatment of inflammatory and infectious skin conditions, reflecting a longstanding ethnopharmacological legacy. These treatments often involve the use of infusions, ointments, poultices, or plant-based oils, applied topically to address a range of dermatological ailments, including inflammation, wounds, burns, eczema, and skin infections. For inflammatory skin conditions, such as dermatitis, redness, and localized swelling, *Hypericum species* has been traditionally used to soothe and repair damaged skin [4]. Similarly, *Salvia fruticosa* and *Satureja species* have been used as antiseptic washes or compresses, particularly effective in reducing minor inflammation and irritation, owing to their essential oil content rich in terpenes and phenolic compounds [5,6]. In the case of wound care and skin trauma, plants such as *Cistus creticus* and *Cotinus coggygria* have been applied for their astringent and antimicrobial properties, assisting in wound drying, infection control, and tissue regeneration. Their traditional use is supported by their high tannin content, which contributes to both hemostatic and anti-inflammatory effects [7,8,9]. For eczema, chronic dermatitis, and dry or scaly lesions, *Juniperus oxycedrus* has been valued for its cade oil, traditionally used to manage persistent skin conditions [10]. Likewise, *Sambucus nigra* has been applied in various forms to accelerate wound healing and ameliorate skin inflammation [11]. Some species, such as *Psoralea bituminosa*, have been referenced in traditional practices for the management of hair loss [12]. Other taxa, including *Teucrium chamaedrys* and *Dittrichia graveolens*, have been noted in regional folk medicine for their topical use in skin eruptions, minor infections, or ulcerations [13,14,15]. Collectively, these plants demonstrate a broad ethnopharmacological relevance in managing inflammatory, infectious, and chronic skin conditions, with their traditional uses closely aligned with the bioactive compounds identified in modern phytochemical research. This heritage supports their further study in the context of natural product-based dermatological therapies.

Inflammation and oxidative stress are key pathophysiological mechanisms that contribute to the onset and progression of many chronic diseases [16,17]. Prolonged inflammatory response, combined with the overproduction of reactive oxygen species (ROS), leads to cellular damage and disruption of the body’s homeostasis. The use of herbal medicinal extracts is a therapeutic approach, as their bioactive antioxidant components can reduce oxidative stress and regulate the inflammatory response, for example by inhibiting enzymes such as COX and LOX, contributing to the prevention and treatment of diseases associated with chronic inflammation [18].

Furthermore, the regulation of enzymes related to skin health, such as tyrosinase and collagenase, is crucial. Tyrosinase controls melanin synthesis and is therefore associated with hypo- or hyperpigmentation disorders, while collagenase breaks down collagen, causing degradation of the extracellular matrix and loss of skin elasticity and strength [19]. Plant extracts exert a protective effect by inhibiting these enzymes and enhancing the maintenance of skin structure and function, particularly in the context of dermatological disorders.

The present study focuses on the analysis of the phytochemical profile and the pharmacological evaluation of seventeen Balkan herbal species with ethnopharmacological interest, using antioxidant and anti-inflammatory assays. Based on the study by Tsioutsiou et al. [13], along with additional literature regarding phytochemical content and biological properties, plant species belonging to the Balkan flora were selected and prioritized for further investigation due to their notable bioactive potential, particularly in relation to their traditional medicinal use and reported pharmacological properties. A total of fifty-four extracts derived from seventeen different plant species were initially screened in the first set of assays. Due to the large number of samples, only the most active extracts were selected to proceed to subsequent assays. Based on their traditional use and supported by the findings of this study along with the existing literature, the most promising plant extracts have been identified for future investigation against skin diseases associated with inflammation or oxidative stress. The study was structured to compare the bioactivity of all extracts at specific concentrations in a high-throughput screening context. High-Performance Thin-Layer Chromatography (HPTLC) was used to analyze the phytochemical profile of the extracts, while HPTLC-DPPH bioautography was conducted in order to assess the presence of antioxidant compounds. Colorimetric assays, such as Folin–Ciocalteu and aluminum chloride, were utilized to quantify phenolics and flavonoids. Further in vitro assays were performed, such as DPPH radical scavenging activity, iron-chelation capacity (ferrozine assay), tyrosinase and collagenase inhibition (as an indicator of possible effect on the skin), and lipoxygenase and cyclooxygenase inhibitory activity (indicative of their anti-inflammatory activity). Finally, carrageenan-induced paw edema served as a model of in vivo anti-inflammatory activity of five selective extracts [20]. The in vitro and in vivo assays are employed to identify antioxidant and anti-inflammatory agents with potential relevance to skin health, as further supported by enzyme inhibition studies targeting skin-related biological pathways. The insights obtained from this study could contribute to the validation of the traditional medicinal uses of these plant extracts and support the discovery of novel natural compounds with significant biological activity.

## 2. Results and Discussion

### 2.1. Extraction, High Performance Thin Layer Chromatography (HPTLC) Profiling and DPPH Bioautography

After ultrasonic extraction, three extracts were obtained from each plant species using different solvents of increasing polarity: dichloromethane (D), methanol (M), and a water–methanol mixture (1:1) (WM). All extracts that occurred were dried using evaporation under vacuum and lyophilization methods, while the corresponding percentage yields were calculated. Among various factors, polarity is generally considered the most critical when extraction time and temperature are held constant [21]. Information on the plant species examined, including its botanical name, family, geographic origin, plant material used, and unique assigned code, as well as the organic solvents employed for extraction, is presented in Table 8.

As indicated in Table 1, the extraction yields of the dichloromethane extracts are lower than those of the methanolic and hydroalcoholic extracts. The yields of the dichloromethane extracts do not exceed 9%, whereas several methanol and hydroalcoholic extracts demonstrated yields above 10%. The differences in the extract yields from the tested plant material might be ascribed to the different availability of extractable components that depend on the varied chemical composition of plants [22]. In order to have a first estimate of the phytochemical content and the presence of antioxidant components, High Performance Thin Layer Chromatography (HPTLC) profiling, as well as DPPH bioautography, were used. After observing the HPTLC chromatograms of the extracts, it is obvious that the chromatograms of dichloromethane extracts obtained by different plant species have only some common spots, while the chromatograms of methanolic and hydroalcoholic solutions show greater similarity for each herbal material. In more detail, regarding the dichloromethane extracts, the absorption of chromatograms at 366 nm revealed characteristic orange-red and violet spots corresponding to chlorophylls, which are localized in the aboveground parts of the plants. In addition, after spraying with vanillin sulfate in visible light, purple spots correspond to fatty substances and terpenes. In addition, common spots are observed at Rf values of 0.2 and 0.6 in the chromatograms of methanolic and hydromethanolic extracts, which showed absorption at 254 nm and 366 nm. The blue coloration of the above-mentioned spots at 366 nm wavelength may indicate the presence of phenolic compounds, which do not appear after spraying with vanillin sulphate. At the same time, the appearance of orange-yellow coloration in the visible after spraying with vanillin sulphate may suggest the presence of flavonoids. The majority of methanolic and hydroalcoholic extracts are characterized by the presence of flavonoids of different classes (flavonols, flavones, flavanols, flavanones), tannins and other phenolic derivatives (phenylpropanoids). Moreover, in the case of the hydroalcoholic extracts’ chromatograms, at Rf > 0.9 and after spraying with vanillin sulphate, grey spots are visible, indicating the presence of sugars.

Among the dichloromethane extracts, the extract of *H. empetrifolium* (D) showed the highest yield, (8.7%) probably due to unpolar compounds like hyperforins, lipid derivatives and fatty acids [23]. In addition, the methanolic extract of *J. Regia* (M) was produced in the highest yield, (22.1%) because of phenyl propanoids (neolignanes) and flavonoid glucosides [24], while *T. chamaedrys* WM demonstrated the highest yield (20.3%) among the hydroalcoholic extracts owing to phenyl-ethanoid glycosides, iridoides, flavonoids and other phenolic glycosidic [25] (Table 1).

Furthermore, the DPPH-bioautography confirmed that all methanolic and hydroalcoholic extracts containing phenolic compounds possess significant free radical scavenging activity, highlighting the categories of natural products that possess activity against oxidative stress (see Figure S1 in Supplementary Materials).

### 2.2. Evaluation of the Total Phenolic Content (TPC) and Total Flavonoid Content (TFC)

Total phenolic content was estimated by using the Folin–Ciocalteu assay, in which phenolic compounds react via a complex redox reaction with the specific reagent [26]. Phenolic compounds are the most abundant group of phytochemicals and contribute significantly to the antioxidant capacity of plants and plant-derived products [27]. Based on Table 2, it is obvious that dichloromethane extracts have a lower TPC compared to the corresponding methanolic and hydroalcoholic extracts. Among them, *S. thymbra* D has the highest TPC value, 59.2 mg GAE/g dw. Overall, the highest amount of polyphenols was observed in *C. coggygria* M and *C. coggygria* WM extracts (168.0 mg GAE/g dw and 123.2 mg GAE/g dw, respectively). Other extracts that showed high polyphenol content (≥99 mg GAE/g dw) were *H. empetrifolium* WM, *J. oxycedrus* WM, *O. dictamnus* WM, *S. triloba* M & WM, *S. thymbra* M & WM, *S. sediforme* M & WM, *T. chamaedrys* M & WM, *T. capitata* M (109.6, 104.8, 102.2, 108.7 & 99.8, 104.3 & 121.1, 106.1 & 95.1, 111.8 & 119.1 and 99.8 mg GAE/g dw, respectively).

The comparative analysis of total phenolic content (TPC) across seventeen different plant taxa revealed both alignment with and divergence from previously published data. For instance, the hydromethanolic extract of *C. salonitana* (38.0 mg GAE/g dw) closely corresponds to the 34.4 mg GAE/g dw reported by Buzhala et al. [28], while in *C. creticus* ssp. *creticus*, the 50% methanol–water extract yielded 169.8 mg GAE/g dw, which is consistent with the 157.17 mg GAE/g dw value reported by Palaiogiannis et al. [29]. In *C. coggygria*, the TPC of the methanolic extract (167.8 ± 10.7 mg GAE/g dw) was lower than the value reported in the literature (208 mg GAE/g dw [30]), a trend similarly observed in the methanolic extract of *D. graveolens*, which yielded 30.6 mg GAE/g dw compared to 86.19 mg GAE/g dw reported by Boudkhili et al. [31]. For *H. empetrifolium*, the hydromethanolic extract exhibited a TPC of 109.6 ± 3.0 mg GAE/g dw, which slightly exceeded the value reported for the aqueous extract (99.84 mg GAE/g dw [23]). In *J. regia*, the methanolic extract yielded a TPC of 66.6 mg GAE/g dw, closely aligning with the value reported for kernels (67.65 mg GAE/g dw [32]), while the hydromethanolic extract produced a higher TPC of 99.1 mg GAE/g dw, exceeding the range reported in the literature for other plant parts (32.61–74.08 mg GAE/g dw [33]). Similarly, the methanolic and hydromethanolic extracts of *J. oxycedrus* yielded TPC of 92.6 and 104.8 mg GAE/g dw, respectively, which were lower than the value of the methanolic extract reported by Bellik and Mekhoukh [34] (~320 mg GAE/g dw) but higher than the value of the hydromethanolic extract (79.38 mg GAE/g dw) reported by Kachmar et al. [35]. In the case of *O. dictamnus*, the TPC of the methanolic extract (74.3 mg GAE/g dw) was substantially lower than the values reported for cultivated samples (172 and 200 mg GAE/g dw [36,37]). Extracts from *P. nigra* exhibited TPC values ranging from 57.0 (hydroalcoholic) to 60.8 mg GAE/g dw (methanolic), which were markedly higher than those reported in the literature for needles and bark (12.0–18.5 mg GAE/g dw [38]; 18.46 mg GAE/g dw [39]). *S. fruticosa* TPC of the methanol extract (108.7 mg GAE/g dw) was lower than the values reported in previous studies, including those by Dawra et al. [40], Kalpoutzakis et al. [37], and Duletić-Laušević et al. [41]. The TPC value of the *S. nigra* hydroalcoholic extract (47.1 mg GAE/g dw) was higher than the range reported by Papagrigoriou et al. [42] (86.75–306.37 mg GAE/100 g dw), and within the range reported by Mihaylova et al. [43] (34.21–47.46 mg GAE/g dw). The TPC of the methanolic extracts of *S. thymbra* (104.3 mg GAE/g dw) and *S. sediforme* (106.1 mg GAE/g dw) were notably higher than those reported in the literature, with *S. thymbra* exceeding the range of 18.32 to 38.79 and *S. sediforme* surpassing the 78.24 mg GAE/g dw reported by Trabsa et al. [44]. Similarly, the TPC values of *T. chamaedrys* methanolic (111.8 mg GAE/g dw) and hydroalcoholic (119.1 mg GAE/g dw) extracts were lower than those reported for aqueous extracts (136.75–208.17 mg GAE/g dw) from various plant parts and methanolic extracts (144–175.46 mg GAE/g dw) as documented by Stanković et al. [45]. The TPC values of *T. capitata* obtained in this study for the methanolic (99.8 ± 0.8 mg GAE/g dw) and hydroalcoholic (78.9 ± 0.5 mg GAE/g dw) extracts were lower than the corresponding values reported in the literature for methanolic (127.52 mg GAE/g dw) and aqueous (94.57 mg GAE/g dw) extracts [46]. Overall, these findings confirm that solvent polarity critically influences phenolic recovery, with methanol and methanol–water mixtures outperforming less polar alternatives like dichloromethane.

Total flavonoid content was measured by the aluminium chloride colorimetric assay and is reported in Table 3. Dichloromethane extracts have the lowest (almost none) total flavonoid content, while the methanolic and hydroalcoholic extracts showed very similar content. The highest TFC was found for the plant extract of *J. regia* M & WM (58.3 & 54.8 mg QUE/g dw), followed by *S. nigra* M & WM, *S. triloba* M & WM, *T. chamaedrys* M and *D. graveolens* WΜ (45.8 & 42.5, 40.1 & 45.5, 42.9 and 41.1 mg QUE/g dw, respectively).

The total flavonoid content (TFC) values obtained in this study generally show good agreement with, or surpass, those reported in the literature across the investigated plant species, highlighting the influence of extraction solvent on flavonoid recovery. In *C. creticus* ssp. *creticus*, the TFC of the methanolic extract was 18.5 mg QE/g dw, markedly exceeding the 2.38 mg QE/g dw reported by Palaiogiannis et al. [29]. In *D. graveolens*, TFC values (21.3 mg QE/g dw) were consistent with previously reported value of 9.72 mg QE/g dw from Boudkhili et al. [31]. The TFC for the methanolic extract of *H. empetrifolium* (30.4 mg QE/g dw) was well above the reported values (2.33–7.23 mg QE/g dw [47]). For *J. regia* leaves, methanolic extract values reached 58.3 mg QE/g dw, although these were considerably lower than the exceptionally high flavonoid content of 391 mg QE/g dw reported in kernel methanolic extracts [32]. In *J. oxycedrus*, the TFC value reported in the literature for the methanolic extract (24 mg QE/g dw [34]) was substantially higher than the corresponding value obtained in the present study (ND), indicating a lower flavonoid extraction efficiency under the experimental conditions applied. For *S. fruticosa*, the TFC values obtained from both the methanolic (40.1 mg QE/g dw) and hydromethanolic (45.5 mg QE/g dw) extracts were approximately twofold higher than those reported by Duletić-Laušević et al. [41]. Similarly, the hydroalcoholic extract of *S. nigra* exhibited a TFC value of 42.5 mg QE/g dw, markedly exceeding the literature range of 11.69–16.18 mg QE/g dw reported by Mihaylova et al. [43]. *S. sediforme* exhibited a TFC value of 22.5 mg QE/g dw, which is in close agreement with the 26.66 mg QE/g dw reported by Ertaş et al. [48]. Collectively, the results emphasize the critical role of solvent polarity—particularly the effectiveness of methanol and methanol–water mixtures—in enhancing flavonoid extraction across diverse plant species.

### 2.3. In Vitro Biological Assays

#### 2.3.1. Evaluation of the Free Radical Scavenging Activity (DPPH)

The DPPH radical scavenging activities of the herbal extracts are shown in Table 4. A primary screen was conducted at 200 μg/mL, after which the most potent extracts were further evaluated at 100 and 50 μg/mL. Instead of calculating IC_50_ values, the study focused on a comparative evaluation of all fifty-four plant extracts. Gallic acid was used as a reference compound with IC_50_: 4.2 μg/mL. Dichloromethane extracts exhibited weaker radical scavenging activity compared to methanolic and hydroalcoholic extracts. This result is attributed to the presence of non-polar compounds, which show a weaker ability to neutralize the DPPH free radical, compared to the polar compounds (phenols and flavonoids) that mainly appear in methanolic and hydroalcoholic extracts. Among the methanolic extracts, the highest radical scavenging activity at 200 μg/mL was observed from the extracts of *C. coggygria* M (96.4%), *S. sediforme* M (94.8%), *C. creticus* M (93.7%), *J. oxycedrus* M (93.5%) and *T. chamaedrys* M (92.3%). Regarding the hydroalcoholic extracts, the highest radical scavenging activity at 200 μg/mL was shown by the extracts of *C. coggygria* WM (95.8%), *S. sediforme* WM (94.8%), *C. creticus* WM (94.3%), *H. empetrifolium* WM (93.0%), *S. triloba* WM (92.8%) and *J. oxycedrus* WM (92.7%).

A comparison of DPPH radical scavenging activity between literature values and the experimental results of this study reveals both consistencies and notable differences across various plant species. For *C. salonitana*, the hydromethanol extract showed comparable inhibition (17.9% at 200 μg/mL) with that of the literature value of 18.3% [28]. *C. creticus* ssp. *creticus* displayed strong activity, with methanol extracts achieving 93.7% inhibition, closely matching the literature’s 100% at 200 μg/mL [37]. *C. coggygria* demonstrated very high activity in this study as well, with 95.1% inhibition at 100 μg/mL, aligning with the literature-reported 95% at 125 μg/mL, indicating antioxidant potential [49]. In *D. graveolens*, the methanol (52.7%) and water–methanol (74.0%) extracts at 200 μg/mL showed lower activity than literature values; Souri and Shakeri reported 85–86% inhibition at 100 mg/mL for the hydroalcoholic extract [50], while Boudkhili et al. noted 89.46% for the methanolic extract at 250 μg/mL [31]. For *J. regia*, the methanolic extract demonstrated comparable activity, with 59.8% inhibition at 100 μg/mL, aligning closely with Das et al., who reported 59.16% at the same concentration and an IC_50_ of 78.77 μg/mL, indicating reproducible activity results and antioxidant potential [32]. In *J. oxycedrus*, the methanolic and hydromethanolic extracts exhibited higher activity (IC_50_ < 50 μg/mL) than the IC_50_ of 320 mg/mL reported by Bellik and Mekhoukh [34], but lower than the aqueous extracts from Kachmar et al. (IC_50_ 9.39–16.05 μg/mL) [35] and El Jemli et al. (IC_50_ 17.91 μg/mL) [51]. The methanolic extract of *O. dictamnus* showed 90% inhibition at 200 μg/mL, closely aligning with Kalpoutzakis et al. (94%) [37], while *P. nigra* exhibited 86.5% inhibition at 200 μg/mL, similar to the 87.2% reported by Yesil-Celiktas et al. at 250 μg/mL, indicating consistent potential antioxidant profiles [52]. For *S. fruticosa*, the methanolic extract showed significantly lower Teramachi inhibition at 50 μg/mL (39.7%) compared to Dawra et al. (76.1%) [40], but exhibited 90.3% inhibition at 200 μg/mL, in close agreement with Kalpoutzakis et al. (100%) [37], suggesting concentration-dependent consistency. The methanolic extract of *S. sediforme* showed substantially lower activity (IC_50_ > 50 μg/mL) than the IC_50_ of 9.07 μg/mL reported by Ertaş et al. [48], while *T. chamaedrys* also demonstrated lower activity (IC_50_ ≈ 70 μg/mL) compared to the 24.51–47.07 μg/mL range reported by Stanković et al. [45].

#### 2.3.2. Εvaluation of Tyrosinase and Collagenase Inhibition Activity

The methanolic and the hydroalcoholic extracts were evaluated for their tyrosinase inhibitory activity, and the results are expressed as percentage (%) of tyrosinase inhibition at 300 μg/mL (Table 5). Instead of calculating IC_50_ values, the study focused on a comparative evaluation of all thirty-six plant extracts. Dichloromethane extracts were not tested since, after mixing their solutions with the enzyme and substrate buffer solutions, insoluble precipitates/suspensions were formed. Kojic acid (IC_50_: 2 μg/mL) and the methanolic extract of liquorice roots (IC_50_: 5 μg/mL) were used as reference inhibitors. The majority of the evaluated extracts (>80%) showed high anti-tyrosinase activity. The strongest inhibitory activity was observed by the methanolic extract of *C. creticus* M and hydroalcoholic extract of *C. coggygria* WM (both 94.2%), followed by the hydroalcoholic extract of *C. creticus* WM (93.3%) and methanolic extracts of *P. nigra* M (92.1%), *C. coggygria* M (83.1%), *S. sediforme* M (81.4%) and *C. salonitana* Μ (80.7%). The plant extracts of *E. graecus* roots, *J. regia* and *P. bituminosa* did not show any inhibitory activity at the tested concentration.

Furthermore, Table 5 also summarizes the results of collagenase inhibition. The values are presented as a percentage (%) of collagenase inhibition at 100 μg/mL. It is noteworthy that the hydroalcoholic extract of *O. dictamnus* WM showed the highest anti-collagenase activity (100.0%). Overall, most extracts exhibited moderate inhibitory activity, with only seven extracts showing strong activity (>85%); *C. creticus* (96.1% M & 94.7% WM), *C. coggygria* (86.6% M & 94.8% WM) and *S. sediforme* (83.6% M & 89.6% WM). The extracts, which possessed very low (<15%) or no activity, were the methanolic and hydroalcoholic extracts of *E. graecus* roots (8.0% M & ND WM), as well as the methanolic extracts obtained from *E. graecus* aerial parts (9.8%) and *J. regia* (11.7%). As a reference compound, phosphoramidon disodium salt was used, which exhibited an IC_50_ value of 3.75 μg/mL.

In order to select the most promising extracts for further investigation, those demonstrating the highest efficacy in the aforementioned assays were subjected to in vitro and in vivo studies to further evaluate their antioxidant and anti-inflammatory properties. Dichloromethane extracts were omitted due to their insolubility in the solvent systems employed in the biological assays. Among the selected plant extracts are the methanolic extracts of *C. salonitana* M, *C. creticus* M, *C. coggygria* M, *E. graecus* aerial parts M and roots M, *S. sediforme* M, *T. capitata* M and *P. bituminosa* M, as well as the hydroalcoholic extracts of *D. graveolens* WM, *H. empetrifolium* WM, *J. regia* WM, *J. oxycedrus* WM, *O. dictamnus* WM, *P. nigra* WM, *P. bituminosa* WM, *S. triloba* WM, *S. nigra* WM, *S. thymbra* WM and *T. chamaedrys* WM.

In the present study, several plant extracts demonstrated varying degrees of tyrosinase inhibitory activity in comparison to the existing literature. The methanolic extract of *C. creticus* exhibited 94.2% tyrosinase inhibition at 300 μg/mL, compared to 63% reported in the literature at the same concentration [53]. Similarly, the methanolic extract of *C. coggygria* demonstrated 83.1% inhibition at 300 μg/mL, markedly higher than the 43% reported at the same concentration in the previous literature [53]. Furthermore, ethanolic extracts examined in earlier studies showed lower inhibition, ranging from 21.98% to 27.59% at 133 μg/mL and from 46.20% to 64.43% at 666 μg/mL [54,55]. For *H. empetrifolium*, the methanolic extract exhibited moderate activity, with 37.6% inhibition at 300 μg/mL, consistent with literature findings where a methanolic extract showed 5.35% inhibition at 100 μg/mL [56], and ethanolic extracts demonstrated 40.86% to 49.91% inhibition at 200 μg/mL [47]. In the case of *J. regia*, the methanolic extract in this study did not exhibit any measurable anti-tyrosinase activity, contrasting with the findings of Bourais et al., who reported IC_50_ values ranging from 51.38 to 87.82 μg/mL [57]. However, this observation is consistent with Uysal et al., who reported only 3–6% inhibition for methanolic and aqueous extracts at 2 mg/mL [58]. The methanolic extract of *O. dictamnus* exhibited 44.2% tyrosinase inhibitory activity at 300 μg/mL, which is in partial agreement with previously reported data showing 11.69% inhibition at 100 μg/mL [56], suggesting comparable efficacy when considering concentration differences. In the case of *P. bituminosa*, the methanolic extract showed limited inhibition (9.9% at 300 μg/mL), whereas Sklirou et al. reported a moderately higher activity of 21.56% at 100 μg/mL [56]. The methanolic extract of *S. fruticosa* exhibited 29.4% inhibition at 300 μg/mL in this study, which does not align with the 44.37% reported by Sklirou et al. at 100 μg/mL [56]. Similarly, the anti-tyrosinase activity of *S. nigra* observed in this study (44.9%) differs from the previous literature: Sklirou et al. reported no inhibitory activity at 100 μg/mL [56], while Tundis et al. reported IC_50_ values of 62.5 μg/mL for the flowers and 204.5 μg/mL for the leaves [59], highlighting the influence of plant part and extract composition. Both *S. thymbra* and *S. sediforme* were also examined in the study by Sklirou et al., yet the results differ from those observed here [56]. The methanolic extract of *S. thymbra* showed 37.5% inhibition at 300 μg/mL, whereas Sklirou et al. reported no activity at 100 μg/mL. For *S. sediforme*, the present study reported 81.4% inhibition at 300 μg/mL, exceeding the 70.55% reported by Sklirou et al. at 100 μg/mL. Another study confirmed this trend, reporting 70.55% inhibition at 300 μg/mL and 40% at 75 μg/mL, supporting the idea of concentration-dependent activity [53]. The methanolic extract of *T. chamaedrys* exhibited 37.6% inhibition at 300 μg/mL in this study, compared to 6.55% at 100 μg/mL reported by Sklirou et al. [56].

Among the selected plants, only *C. coggygria* and *S. fruticosa* have been previously studied for their activity against collagenase, specifically through the investigation of their ethanolic extracts. In the present study, the methanolic extract of *C. coggygria* exhibited strong collagenase inhibitory activity, with 94.8% inhibition at 100 μg/mL. In comparison, the study by Senol Deniz et al. reported that the ethanolic extract at a concentration of 666 μg/mL showed collagenase inhibition ranging from 46.51% to 55.30%, while at 133 μg/mL, the activity ranged from not detected (ND) to 20.08% [54,55]. Additional studies have demonstrated that at a higher concentration of 2 mg/mL, the ethanolic extract displayed either moderate activity (52.52%) or very strong inhibition, ranging from 99.62% to 99.88% [60,61]. Regarding *S. fruticosa*, Senol Deniz, Orhan, and Duman reported no collagenase inhibitory activity for the ethanolic extract at 666 μg/mL, in contrast to the present findings, where the methanolic extract exhibited significant inhibition, reaching 59.3% at the same concentration [55].

It should be noted, however, that the above-mentioned interesting activities of several extracts on these enzymes were performed as a preliminary screening of total contents and as such are subjected to limitations such as non-specificity and potential overestimation, while they also may be subjected to translational limitations to human systems.

#### 2.3.3. Εvaluation of Fe^2+^ Chelating Capacity

Iron ions contribute to the production of oxidative stress through their catalytic role in the decomposition of hydrogen peroxide (Fenton reaction), leading to the formation of highly reactive hydroxyl radicals (**·**OH). Thus, the chelation of excess free iron is important to limit the damage occurring from oxidative stress [62,63]. Ferrozine produces a violet-colored complex with Fe^2+^. This complex formation is interrupted in the presence of chelating agents, and as a result, the violet color of the complex (absorption) is decreased [64]. The IC_50_ values and the % Free Fe^2+^ at 200 μg/mL are given in Table 6. The lower the amount of free iron, the more effective the chelate binding by the plant extract. Consequently, increased residual free iron correlates with higher percentages in Table 6, indicating decreased plant extract efficacy and increased IC_50_ values. The results demonstrated that the formation of Fe(II)–ferrozine complex was decreased in the presence of most of the tested plant extracts. In more detail, the chelation ability of extracts obtained from *H. empetrifolium* WM (IC_50_: 5.0 μg/mL), *S. sediforme* M (IC_50_: 26.0 μg/mL) and *C. coggygria* M (IC_50_: 37.0 μg/mL) were the most significant. The findings of this assay corroborate the previously observed remarkable content of antioxidant substances in these three extracts, as evidenced by the TPC and DPPH assays. The results were compared with EDTA used as a standard.

The comparison between the literature and the experimental results reveals both consistencies and discrepancies across the plant species evaluated for their iron chelating capacity. *C. creticus* ethanolic extract showed modest Fe^2+^ chelation in the literature (<30% at 200 μg/mL) [65], which aligns with the present study’s finding of 30% free Fe^2+^ at 200 μg/mL using a methanolic extract, indicating similar moderate activity. The methanolic extract of *C. coggygria* demonstrated significantly stronger iron chelating activity in the literature, with 78% Fe^2+^ chelation at 20 μg/mL [66], while the current study reported 10.8% free Fe^2+^ at 200 μg/mL, also indicative of high chelating capacity. However, Senol Deniz, Orhan, and Duman reported negligible activity for the hydroethanolic extract at a much higher concentration (5 mg/mL), with only 7.17% Fe^2+^ chelation, underscoring the influence of extraction solvent and methodology [55]. Similarly, the methanolic extract of *D. graveolens* in the literature showed moderate chelation (63.43% [67] and 70% [68] at 2 mg/mL; IC_50_: 194–205 μg/mL [69]), while the hydromethanolic extract in the present study displayed markedly greater efficacy, with only 18.8% free Fe^2+^ at 200 μg/mL and an IC_50_ of 25.0 μg/mL. For *J. regia*, methanolic extracts have shown wide variability in the literature (IC_50_ values from 43.0 to 388.61 μg/mL [70,71,72]), and the current results (49.4% free Fe^2+^, IC_50_ 201.3 μg/mL) fall within this range, indicating moderate agreement. The hydromethanolic extract of *P. nigra* also exhibited greater chelation efficiency in this study (IC_50_: 118.5 μg/mL, 19.9% free Fe^2+^) compared to the literature data (53.2% chelation at 375 μg/mL) [73]. In contrast, *S. fruticosa* showed weak activity in the literature (12.73% and 25% chelation at 5 mg/mL and 8.37 mg/mL, respectively [55,74]), whereas this study reported stronger activity (14% free Fe^2+^, IC_50_ 121.1 μg/mL), indicating a notable discrepancy. *S. nigra* methanolic extract had an IC_50_ of 133.6 μg/mL according to Azari et al. [75], which aligns well with the IC_50_ of 115.5 μg/mL obtained for the hydromethanolic extract in the present study. For *S. sediforme*, the literature reported an EC_50_ of 0.985 mg/mL [44], while the current study observed a much lower IC_50_ of 25 μg/mL, indicating that extracts appear more potent under the present experimental conditions. Similarly, *T. capitata* showed > 20% and 80% Fe^2+^ chelation in the literature at 250 mg/L and 1 g/L [76], respectively, whereas the present study reported 31.6% free Fe^2+^ at 200 μg/mL, suggesting strong activity at a substantially lower dose under the current experimental conditions.

#### 2.3.4. Εvaluation of Lipoxygenase Inhibitory Activity

The initial evaluation of the extracts’ anti-inflammatory effect focused on their ability to inhibit soybean lipoxygenase (LOX-3) in vitro. Lipoxygenase (LOX) converts arachidonic, linoleic, and other polyunsaturated fatty acids (PUFAs) into biologically active metabolites that are involved in inflammatory and immune responses [77]. The anti-inflammatory capacity of the plant extracts on LOX-3 was evaluated at a concentration of 200 μg/mL, while the most promising samples were further investigated for their efficacy at lower concentrations. The results are shown in Table 7 as IC_50_ values or % inhibition of 200 μg/mL. Among the plant extracts tested, the most active, showing IC_50_ values below 100 μg/mL, were the methanolic extracts obtained from *T. capitata* M, *S. sediforme* M and *C. coggygria* M (IC_50_: 28.0, 39.4 and <100 μg/mL, respectively). The bioactivity of the plant extracts was compared with that of standard reference compound ferulic acid, which exhibited an IC_50_ value of 25.6 μg/mL. However, the activity of plant extracts did not reach the effect of the known NSAID naproxen (IC_50_ value of 5.76 μg/mL).

When comparing LOX inhibition data from the literature with the experimental results of this study, both consistencies and discrepancies are evident. *C. coggygria* has been previously reported to exhibit 14.6% inhibition of lipoxygenase (LOX) activity at a concentration of 2 mg/mL using an ethanolic extract [60]. In contrast, the methanolic extract examined under the experimental conditions of the present study, demonstrated a markedly higher inhibitory potential, achieving 99.4% LOX inhibition at just 200 μg/mL. Furthermore, Marčetić et al. investigated the ethyl acetate fraction of an acetone extract of *C. coggygria* and reported substantial LOX inhibitory activity, with an IC_50_ value of 72.05 μg/mL [78], supporting the strong anti-inflammatory potential of specific solvent fractions. In *H. empetrifolium*, a dichloromethane extract has been reported in the literature to exhibit almost complete LOX inhibition (99.7%) at a concentration of 20 μg/mL, suggesting a very low IC_50_ value [79]. In comparison, the hydromethanolic extract evaluated in the present study demonstrated reduced potency, achieving 88.8% inhibition at 200 μg/mL, with an estimated IC_50_ of approximately 103 μg/mL. This discrepancy likely reflects the impact of extraction solvent polarity on the yield and profile of bioactive constituents, emphasizing the solvent-dependent nature of the anti-inflammatory activity. *J. regia* has also been reported to exhibit significantly higher LOX inhibitory activity in the literature, with methanolic extracts showing IC_50_ values ranging from 28.38 to 30.56 μg/mL [57]. In contrast, the extract analyzed in the present study demonstrated 60.1% inhibition at 200 μg/mL, corresponding to a markedly higher IC_50_ of 192.6 μg/mL. The hydromethanolic extract of *J. oxycedrus* in this study exhibited superior LOX inhibitory activity (67.3% at 200 μg/mL; IC_50_ ~147.3 μg/mL) compared to the aqueous extract reported in the literature (<50% inhibition at 240 μg/mL) [80], likely due to improved recovery of active compounds through the presence of methanol in the solvent extraction. In *S. fruticosa*, the ethanol extract reported in the literature exhibited 47.57% LOX inhibition at a concentration of 2 mg/mL [60], whereas the hydromethanolic extract evaluated in the present study achieved a comparable level of inhibition (~47.7%) at only 200 μg/mL. Regarding *S. nigra*, Deniz et al. reported minimal LOX inhibitory activity, with an IC_50_ exceeding 500 μg/mL [81]; consistently, the hydromethanolic extract in the present study exhibited only ~12.9% inhibition at 200 μg/mL, confirming the weak LOX-inhibitory potential of this species under both extraction conditions. *T. chamaedrys* has been reported to exhibit notable LOX inhibitory activity in the literature, with a methanolic extract yielding an IC_50_ of 97.38 μg/mL [82]; however, in the present study, the hydromethanolic extract demonstrated substantially lower potency (IC_50_ = 201.3 μg/mL). The methanolic extract of *T. capitata* in the present study demonstrated comparable potency to that reported in the literature for the aqueous extract, exhibiting an IC_50_ of 28.0 μg/mL, versus an IC_50_ of 40 μg/mL for the aqueous extract [83]. Overall, these comparisons reveal that while some plants consistently show low LOX inhibition across studies, many others demonstrate significant variations in percentage inhibition and IC_50_ values between the literature and our findings. Such disparities can be attributed to differences in extraction solvent polarity (which alters the spectrum and yield of active phytochemicals), the specific plant parts or chemotypes analyzed, and methodological conditions of the LOX assay, underscoring the importance of these factors in evaluating and comparing bioactivity results.

#### 2.3.5. Εvaluation of Cyclooxygenase Inhibitory Activity

**Figure 1:**
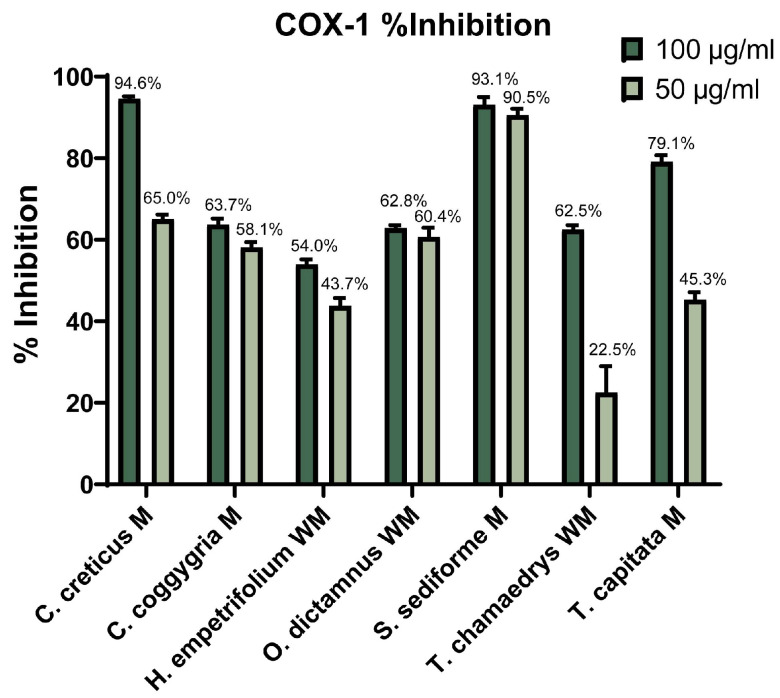
Inhibition (%) of Cycloxygenase-1 by eight Balkan herbal plant extracts at 100 and 50 μg/mL. A concentration of 7.16 μg/mL of the indomethacin exhibited 100% inhibition of COX-1.

**Figure 2:**
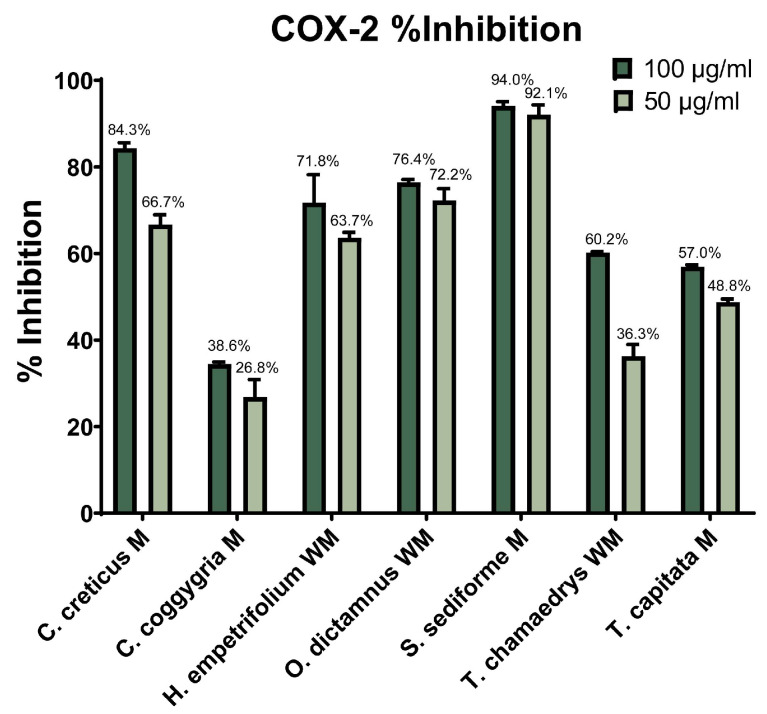
Inhibition (%) of Cycloxygenase-2 by eight Balkan herbal extracts at 100 and 50 μg/mL final concentration. A concentration of 7.16 μg/mL of the indomethacin exhibited 80% inhibition of COX-2.

Cyclooxygenase is an important enzyme in the inflammation process that exists in two isoforms (cyclooxygenase-1 (COX-1) and cyclooxygenase-2 (COX-2)) and participates in the biosynthesis of prostaglandins and leukotrienes from arachidonic acid [84,85]. The evaluation of the seven most promising plant extracts, selected based on the results of the previous in vitro experiments, as well as reported in the literature, for their ability to inhibit COX-1 and COX-2 (at 100 and 50 μg/mL final concentrations) was evaluated and the results are expressed as percentage of inhibition of each cyclooxygenase isoform (Figure 1 and Figure 2). Indomethacin was employed as a reference compound, and at a concentration of 7.16 μg/mL it achieved 100% inhibition of COX-1.

The methanolic extracts of *C. creticus* (94.6%), *S. sediforme* M (93.1%) and *T. capitata* M (79.1%) showed the highest inhibition of COX-1 activity at 100 μg/mL concentration. The rest of the extracts had moderate COX-1 inhibitory activity (<65%) at 100 μg/mL. *S. sediforme* extract was particularly active and its inhibition ability on COX-1 was maintained also at 50 μg/mL concentration, in comparison to *C. creticus* and *T. capitata* that showed considerably reduced activity at 50 μg/mL concentration (Figure 1).

Moreover, regarding the inhibitory activity against Cycloxygenase-2, the most active extracts proved to be the methanolic extracts of *S. sediforme* (94.0%) and *C. creticus* (84.3%), as well as the hydroalcoholic extracts of *O. dictamnus* (76.4%) and *H. empetrifolium* (71.8%). The rest of the extracts had moderate efficacy (<60% inhibition at 100 μg/mL) (Figure 2). As expected, the activity of the extracts on both enzymes is dose-dependent, since the efficiency decreased, or remained the same, after reducing the concentration, e.g., for *S. sediforme*. Indomethacin was employed as a reference compound, and at a concentration of 7.16 μg/mL it achieved 80% inhibition of COX-2. Considering the results of COX-1 vs. COX-2 inhibition, *C. creticus* and *S. sediforme* inhibited both enzymes significantly at a concentration of 100 μg/mL. Extracts exhibiting potent inhibitory effects on the two isoforms of cyclooxygenase consistently demonstrated comparable inhibitory activity also toward lipoxygenase; thus, they may be considered as potentially good anti-inflammatory agents. The extracts of *H. empetrifolium* and *O. dictamnus* displayed greater efficacy on COX-2 enzyme, while the extracts of *C. coggygria* and *T. capitata* were more active against COX-1 enzyme.

Comparative analysis of the inhibitory activity of the extracts against COX-1 and COX-2 isoenzymes revealed different selectivity profiles. The methanolic extracts of *C. creticus* and *S. sediforme* exhibited high and comparable inhibition of both isoenzymes at a concentration of 100 μg/mL, suggesting non-selective inhibition of cyclooxygenase. In contrast, *O. dictamnus* and *H. empetrifolium* extracts showed higher inhibition of COX-2 compared to COX-1, demonstrating a COX-2-selective inhibition profile, while *C. coggygria* and *T. capitata* extracts showed greater inhibitory activity against COX-1 than COX-2, suggesting COX-1-preferential inhibition.

As anticipated, the inhibitory activity of the extracts against both cyclooxygenase enzymes (COX-1 and COX-2) exhibited a dose-dependent relationship; a decrease in concentration generally led to reduced efficacy. An exception was observed with *S. sediforme*, which maintained consistent inhibitory activity across tested concentrations. Previous studies have reported moderate COX inhibition by aqueous extracts of *C. coggygria*. Specifically, Ozsoy et al. determined EC_50_ values of 2.2 mg/mL for COX-1 and 4.3 mg/mL for COX-2 [86]. These findings were corroborated by Antal et al., who reported similar EC_50_ values of 2.21 mg/mL for COX-1 and 4.10 mg/mL for COX-2, indicating a consistent inhibitory profile across studies [87]. The current study demonstrated that the methanolic extract of *C. coggygria* at a concentration of 50 μg/mL, inhibits COX-1 activity by 58.1% and COX-2 activity by 26.8%. Furthermore, Crockett et al. investigated the dichloromethane fruit extract of *H. empetrifolium*, revealing substantial inhibition of COX-1 (85.1%) and moderate inhibition of COX-2 (49.1%) at a concentration of 20 μg/mL [79]. To our knowledge, this study represents the first evaluation of COX inhibitory activity for the remaining tested plant extracts, thereby contributing novel insights into their potential anti-inflammatory properties.

Well-known NSAIDs such as celecoxib (IC_50_ = 0.023 μg/mL for COX-2 and 49.9 μg/mL for COX-1) and meloxicam (IC_50_ = 4.39 μg/mL for COX-2 and 48.4 μg/mL for COX-1) exhibit well-established inhibitory potency and selectivity profiles [88]. Regarding COX-1 inhibition, all plant extracts, with the exception of that of *T. chamaedrys*, demonstrated notable inhibitory activity under the tested experimental conditions, at 50 μg/mL, inhibiting COX-1 by 45–65%while *S. sediforme* extract exhibited 90% inhibition at that concentration rendering it the most active extract and well withing the range of activity of the above mentioned NSAIDs, albeit less active than Indomethacin. Regarding COX-2, 65–70% inhibition was exhibited by *C. creticus*, *H. empetrifolium* and *O. dictamnus* at 50 μg/mL, while *S. sediforme* exhibited the highest inhibitory activity (90%), although relatively lower than the activity of known NSAIDs on COX-2.

### 2.4. In Vivo Biological Assay

#### Carrageenan-Induced Paw Edema Activity

**Figure 3:**
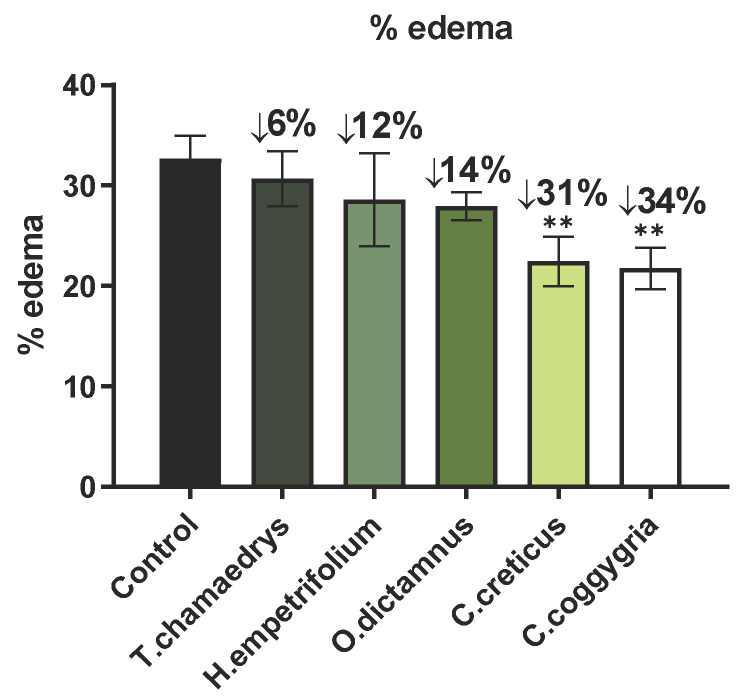
In vivo anti-inflammatory activity of the medicinal plants studied; % edema and its reduction compared to control, of carrageenan-induced mouse paw edema (*n* = 6). ** *p* < 0.01 vs. Control. Arrows (↓) indicate the percentage reduction in edema relative to the control group. Statistical analysis revealed no significant differences among the experimental groups.

Inflammation induced by carrageenan is acute, non-immune, well-researched, and highly reproducible [89]. As a result, the carrageenan-induced paw edema is a well-known model that is widely used for assessing the anti-inflammatory capacity of promising agents [90,91]. The most active plant extracts in vitro were selected for this assay and evaluated at a dose of 0.03 mg/g of body weight (i.p.). The results of the tested extracts are shown in Figure 3 and are expressed as % edema reduction in paw edema 3.5 h after carrageenan injection. It is noteworthy that a statistically significant (*p* < 0.05) reduction in paw edema was observed in the mice that were treated with the extracts from *C. coggygria* and *C. creticus* (34% and 31% edema reduction, respectively). The methanolic extract of *C. creticus* showed the highest inhibitory activity in both COX-1 and COX-2 enzymes in vitro. The suppression of the first phase (after one hour) of the induction of acute inflammation by carrageenan, may be due to inhibition of release of early mediators, such a histamine and serotonin, while suppression of the second phase (after three hours) may be explained by an inhibition of cyclooxygenases [92], and this may hint towards the mode of in vivo activity of these extracts. The results of this in vivo study are in accordance with the in vitro results of inhibition of lipoxygenase and cyclooxygenase, providing further evidence of the anti-inflammatory activity of these extracts.

The results of this in vivo study are in accordance with the in vitro results of inhibition of lipoxygenase and cyclooxygenase, and provide further evidence of the anti-inflammatory activity of these extracts. In the literature, rats are used as the animal model instead of mice; therefore, the doses of the plant extracts administered were considerably higher. Specifically, the group of Marčetić et al. investigated the ethyl acetate fraction of the acetone extract of *C. coggygria* at concentrations of 50 mg/kg and 100 mg/kg, demonstrating a reduction in paw edema by 46.5% and 76.5%, respectively [78]. Regarding *H. empetrifolium*, the methanolic extract at 100 mg/kg showed a 77.40% reduction [93], whereas in the present study, a dose of 30 mg/kg in mice resulted in only an 8.2% reduction. Finally, it has been shown that the aqueous extract of *T. chamaedrys*, at a dose of 200 mg/kg, produced an effect comparable to that of the standard anti-inflammatory drug, indomethacin [14].

Further, oxygen-derived free radicals, particularly those released by phagocytes and known to play a significant role in the inflammation process, activate nuclear factor-kappa B (NF-κB), which in turn triggers the production of inflammatory mediators like cytokines and cyclooxygenase-2 (COX-2). The anti-inflammatory properties observed in these plant extracts might be due to either specific cyclooxygenase inhibition (active compounds acting on their own or in synergism with one another [94]) or a combination of mechanisms, like inhibiting enzymes involved in inflammation, scavenging free radicals, or mimicking the effects of corticosteroids [95]. Finally, absence of a detectable effect in this model does not necessarily mean that the extracts have no anti-inflammatory activity, because the active compound(s) could be interacting at alternative sites (molecular targets) in the complex inflammatory process that are not involved in this specific experimental protocol [96].

## 3. Materials and Methods

### 3.1. Plant Material

Within the framework of the ‘EthnoHerbs’ program, the studied plant materials were collected in the period 2020–2021 from various geographical areas of Greece and Serbia (Table 1). After drying in a shaded area, the plant materials were powdered in a blender to reduce particle size and facilitate extraction. The abbreviations of powdered plant materials and obtained extracts are given in Table 8.

### 3.2. Chemicals and Reagents

All commercially available chemicals are of appropriate purity and purchased from standard sources. Analytical grade methanol (MeOH) and dichloromethane (DCM) for extraction, as well as acetonitrile (ACN), ethyl acetate (EtOAc), Toluene, Acetic Acid (AA), formic acid (FA) sulfuric acid (H_2_SO_4_) and vanillin for HPTLC analysis, ethanol 96% (EtOH) for bioassays and potassium hydroxide (KOH) were purchased from Merck (MerckDarmstadt, Germany). Distilled water was produced from the LaboStar Pro TWF UV ultra-pure water system (Evoqua Water Technologies, Barsbüttel, Germany). For free radical scavenging and total phenolic content assays, Folin–Ciocalteu solution, dimethylsulfoxide (DMSO), sodium carbonate (Na_2_CO_3_), Sodium acetate (SA), aluminum chloride, gallic acid, quercetin and 2,2-diphenyl-1-picrylhydrazyl (DPPH), as well as Collagenase enzyme, were purchased from Sigma-Aldrich (Sigma-Aldrich, Steinheim, Germany). Ovine COX-1 and human recombinant COX-2 enzymes were included in the “COX Inhibitor Screening Assay” kit by Cayman Chemical Co. (Ann Arbor, MI, USA) (Item No. 560131). All reagents used in the tyrosinase and collagenase assays were obtained from Sigma-Aldrich. The phosphate-buffered saline (PBS) used for the tyrosinase assay was prepared with sodium phosphate dibasic (Na_2_HPO_4_) and sodium phosphate monobasic (NaH_2_PO_4_), while the enzyme used was mushroom tyrosinase (Product code: T3824, 25 KU), and the substrate was 3,4-dihydroxy-L-phenylalanine (L-DOPA), with a molecular weight of 197.19. For the collagenase assay, a 10 mM Tris-HCl buffer at pH 7.3 was employed. The enzyme collagenase from *Clostridium histolyticum* (Product code: C9263, 25 mg) was used alongside phosphoramidon disodium salt (Product code: R7385, MW 587.47) as an inhibitor. The fluorogenic peptide substrate specific for MMP2 (MCA-Pro-Leu-Ala-Nva-DNP-Dap-Ala-Arg-NH_2_, Product code: SCP0192) was also sourced from Sigma-Aldrich.

### 3.3. Extraction Procedure (Ultrasound-Assisted Extraction-UAE)

The ultrasonic-assisted extraction device Elma S100H (Elma Schmidbauer GmbH, Singen, Germany), operating at an ultrasonic frequency of 37 kHz, was used. A total of 50 g of dry powdered plant material was sequentially extracted with 500 mL of dichloromethane, 500 mL of methanol, and 500 mL of a hydroalcoholic mixture (H_2_O:MeOH 50:50), in three consecutive cycles for each solvent, with an extraction time of 30 min per cycle and at room temperature. All collected extracts (dichloromethane, methanolic, and hydroalcoholic) were concentrated under reduced pressure using a rotary evaporator, RotaVapor (Büchi Labortechnik AG, Flawil, Switzerland). Subsequently, the hydroalcoholic extracts underwent lyophilization to obtain fully dried residues. The water bath temperature of the RotaVapor was set to 40 °C for the dichloromethane and methanol extracts, and to 50 °C for the hydroalcoholic extracts. Τhe optimum rotation speed is around 250 to 280 rpm (an increase in speed above 200 rpm has a relatively low influence on the evaporation output while rpm above 300 can result in mechanical issues, vibrations and spillage from the heating bath).

The hydroalcoholic extracts were initially dried by vacuum distillation to remove the methanol, followed by lyophilization using a Zirbus freeze dryer (ZIRBUS technology GmbH, Bad Grund (Harz), Germany)—operating at 5 mbar and chamber temperature of −50 °C with an Edwards vacuum pump—to eliminate the remaining water. The stages of the lyophilization process involve initially freezing the extract (−10 °C to −40 °C), applying high vacuum and carefully heating the extract (providing latent heat of ice sublimation). Finally, all the dried extracts were stored at 2–8 °C, where they remained stable until further analysis [97].

### 3.4. Phytochemical Profile

#### 3.4.1. High Performance Thin Layer Chromatography (HPTLC) Profiling

The phytochemical profile of the obtained dichloromethane, methanol and aqueous-alcohol (50:50 *v*/*v* MeOH:H_2_O) extracts was investigated using a High Performance Thin Layer Chromatography (HPTLC) apparatus purchased from CAMAG (Muttenz, Switzerland). More specifically, 20 cm aluminum plates coated with normal-phase silica gel (Merck Silica gel 60 F254) and 20 × 10 cm aluminum plates coated with reverse-phase silica gel (RP-18 F254) were used (Merck, Darmstadt, Germany). All the above extracts were dissolved in the respective solvent at a concentration of 10 mg/mL, while 100 µg of each solution was spotted on the chromatographic plate using an automated sample applicator ATS4 and the chromatograms were developed in an ADC2 automated development chamber with the appropriate mobile phase. More specifically, dichloromethane extracts were analyzed on a normal-phase (NP) aluminum plate with Tol:EtOAc:FA solvent system at a ratio of 70:30:1, while methanolic extracts were analyzed on normal-phase plates with EtOAc:MeOH:H_2_O:FA mobile phases at a ratio of 55:7:5:1. Finally, the hydroalcoholic extracts were analyzed on reverse-phase (RP) plates with H_2_O:ACN:FA solvent system at a ratio of 75:25:1. After obtaining the developed chromatograms, they were photographed in the visible, 254 and 366 nm wavelengths, sprayed with sulphuric vanillin, heated in a heating plate using the TLC Visualizer 2 (CAMAG AG, Muttenz, Switzerland) and re-photographed in the visible and at 366 nm wavelength. The system was operating under the VisionCats 2.2 software (CAMAG AG, Muttenz, Switzerland). HPTLC was employed as a qualitative tool to assess the phytochemical profiles of all extracts. The aim was not quantification, but rather to visualize and compare their chemical composition patterns.

#### 3.4.2. DPPH Bioautography

HPTLC-bioautographic assay refers to the combination of the HPTLC technique with any method for the evaluation of biological properties. In the present work, the HPTLC-DPPH (HPTLC-DPPH-bioautographic method) was applied to search for components with antioxidant activity directly on the chromatographic plate. The plates were derivatised with vanillin/sulfuric acid reagent, each time freshly prepared and subsequently heated on the plate heater. More specifically, the TLC plates developed for the control of phytochemical profile were sprayed with the following mixture of reagents A + B: Solution A: 5% vanillin in methanol, Solution B: 5% H_2_SO_4_ in methanol. Equal volumes of reagents A and B were mixed just before spraying, and the chromatograms were revealed by heating for 3 min at high temperature (>100 °C). Τhey were subsequently immersed in DPPH-methanol solution (0.1 g DPPH in 200 mL MeOH). In this way, the active components of the fractions are detected through the apparent discoloration of their spots (from purple, which is the whole background due to the coloration of the plate by the reagent, to yellow, due to the local neutralization of the free radical of DPPH). The dry extracts were prepared at a concentration of 100 μg/mL, and the reaction time was 30 min. This technique offers reproducible results, requires small amounts of sample and can be used in the bio-guided isolation of bioactive target compounds.

### 3.5. Colorimetric Assays

#### Total Phenolic Content (TPC) and Total Flavonoid Content (TFC)

Total Phenolic Content—TPC

The Folin–Ciocalteu reagent method for determining phenolic content is widely used due to its simplicity and reproducibility [98]. The method is based on the oxidation of phenols with the simultaneous reduction of a phosphomolybdic and phosphotungstic acid solution, resulting in the formation of a phosphomolybdic/phosphotungstic-phenolic complex. The latter turns blue in an alkaline environment and has a maximum absorbance at 765 nm. Phenolic compounds react with the reagent only under alkaline conditions and for this reason the pH is adjusted to 10 with sodium carbonate solution [99]. In 96-well plates, 25 μL of each sample dissolved in DMSO (final concentration of 100 μg/mL), 125 μL of Folin–Ciocalteu reagent (10% in distilled water), and 100 μL of Na_2_CO_3_ solution (7.5% *w*/*v* in distilled water) were placed. After mixing, the samples were incubated for 30 min in the dark at room temperature and then the absorbance was measured at 765 nm using an Infinite m200 Pro TECAN photometer (Tecan Group, Männedorf, Switzerland). Subsequently, a gallic acid reference curve (GAE) was constructed from which the gallic acid equivalents (GAE) for each sample were calculated. From an initial gallic acid solution of 10 mg/mL (100 μg/mL in the well) in DMSO, nine solutions of different concentrations were prepared by successive dilutions, which were used to construct the reference curve. All experiments were performed in triplicate to ensure reproducibility and reliability of the results.

Total Flavonoid Content—TFC

The method is a colorimetric test based on complexation reactions between metals and flavonoids. Specifically, a complex is formed between the Al (III) ion and the carbonyl and hydroxyl groups of flavones and flavonols, which produces a yellow color. To determine the total flavonoid load, 50 µL of each sample dissolved in DMSO (final concentration 400 µg/mL) were first placed in 96-well plates, and then the reagents were added in the following order: 20 µL of AlCl_3_ solution (1.8% *w*/*v* AlCl_3_·6 H_2_O), 160 µL EtOH and finally 20 µL C_2_H_3_NaO_2_ (820.3 mg in 100 mL H_2_O). The plates were incubated for 40 min in the dark at room temperature and then their absorbance was measured at 415 nm using an Infinite m200 Pro TECAN photometer (Tecan Group, Männedorf, Switzerland). Blanks were also used for each sample, which did not contain the AlCl_3_ and sodium carbonate solutions. The quercetin reference curve was used to determine the flavonoid load. Therefore, the absorbance of solutions of known concentration of quercetin (200 µg/mL, 160 µg/L, 120 µg/mL, 80 µg/mL, 40 µg/mL, 10 µg/mL, 5 µg/mL and 2.5 µg/mL) was measured and a reference curve was constructed. All experiments were performed in triplicate to ensure reproducibility and reliability of the results.

### 3.6. In Vitro Biological Assays

#### 3.6.1. Free Radical Scavenging Activity (DPPH)

Evaluation of antioxidant activity was performed according to the following protocol: 12.4 mg DPPH was dissolved in 100 mL of absolute ethanol. The DPPH solution (0.317 mM) was prepared just before the experiments and stored in the dark at room temperature. A primary screen was conducted at 200 μg/mL, after which the most potent extracts were further evaluated at 100 and 50 μg/mL. A total of 10 μL of each sample and 190 μL of DPPH solution were placed in 96-well plates and incubated for 30 min in the dark at room temperature. After incubation, the absorbance was measured at 517 nm using an Infinite m200 Pro TECAN photometer (Tecan Group, Männedorf, Switzerland). Gallic acid was used as a positive control for the inhibition of the DPPH. In addition, each sample was also measured without the addition of DPPH (ethanol only) in order to calculate and subtract the absorbance that may be due to the sample itself. Gallic acid was used as a reference compound. The % inhibition of the DPPH radical for each dilution was calculated using the following formula:

DPPH (%) = [(A − B) − (C − D)]/(A − B) × 100

where A: Control (without sample), B: Blank (without sample and DPPH solution), C: sample, D: blank of the sample (without DPPH solution).

All experiments were performed in triplicate to ensure reproducibility and reliability of the results.

#### 3.6.2. Tyrosinase Inhibition

The ability of the extracts, at concentrations of 300 µg/mL and 100 µg/mL, to inhibit the oxidation of L-DOPA to dopaquinone and subsequently to dopachrome by mushroom tyrosinase was investigated. A total of 40 μL of sample, 80 μL of buffer solution (pH = 6.70 ± 0.02) and 40 μL of tyrosinase [92 U/mL Tyrosinase from mushroom lyophilized powder, ≥1.000 unit/mg solid T3824 (25KU)] were placed in 96-well plates. In the experimental design, each sample was accompanied by a blank prepared without tyrosinase and a control composed of 120 µL buffer solution and 40 µL tyrosinase in the absence of extract. Kojic acid (IC_50_: 2 μg/mL) and the methanolic extract of liquorice roots (IC_50_: 5 μg/mL) were used as reference inhibitors, while 3 repetitions were performed in all samples. After incubation for 5 min at 25 °C, their absorbance was measured at 475 nm using an Infinite m200 Pro TECAN photometer (Tecan Group, Männedorf, Switzerland) [100] and Tyrosinase inhibition was calculated by the following formula:

{[(A − B) − (C − D)]/(A − B)} × 100

where A: Control (without sample), B: Blank (without sample and tyrosinase), C: sample, D: blank of the sample (without tyrosinase).

All experiments were performed in triplicate to ensure reproducibility and reliability of the results.

#### 3.6.3. Collagenase Inhibition

Τhe extracts were tested at concentrations of 100 µg/mL. In black-walled 96-well plates, 25 µL of Tris-HCl buffer (pH = 7.3), 25 µL of sample (dissolved in Tris-HCl buffer) and 25 µL of collagenase from Clostridium histolyticum (100 µg/mL in Tris-HCl buffer) were properly mixed and followed by incubation for 10 min at 37 °C. Then, 25 µL of substrate solution (fluorogenic peptide substrate MMP2 diluted in buffer (55.5 μg/mL)) was added, followed by incubation at 37 °C for 30 min. Finally, fluorescence values were measured at 320 nm excitation and 405 nm emission using an Infinite m200 Pro TECAN photometer (Tecan Group, Männedorf, Switzerland) [100] and calculated using the following formula. Phosphoramidon disodium salt was used as a reference compound and was dissolved in DMSO at a concentration of 1 mg/mL (stock solution). These assays were performed in triplicate using phosphoramidone as a reference inhibitor, and blanks were used for each sample, which did not contain collagenase [101]. Collagenase inhibition was calculated by the following formula:

{[(A − B) − (C − D)]/(A − B)} × 100

where A: Control (without sample), B: Blank (without sample and collagenase), C: sample, D: blank of the sample (without collagenase).

#### 3.6.4. Fe^2+^ Chelating Capacity

The Fe^2+^-chelating capacity was determined by the reduction of the maximum absorption of the Fe^2+^-ferrozine complex. In each well of a 96-well plate, 30 μL of sample plant extract (100–400 μg/mL final concentration, or in some cases lower concentrations for samples with increased activity) were incubated with 30 μL of 20 μM (1.12 μg/mL) Fe^+2^ in ammonium acetate 5% *w*/*v*, pH = 6.9. The reaction started after adding 6 μL of 100 μM ferrozine (56 μg/mL) solution to each well. The absorption is measured at 562 versus 700 nm, after incubation for 10 min at 37 °C. For each sample, a blank was prepared without ferrozine, along with a control that lacked the extract sample. Ethylenediaminetetraacetic acid (EDTA) 100 μM was used as positive control [102]. The most active extracts were also tested in lower concentrations. All results are expressed as the mean values obtained from at least three measurements.

#### 3.6.5. Lipoxygenase Inhibition

Lipoxygenase (LOX) activity was determined using soybean lipoxygenase (250 U/mL) and sodium linoleate (100 µM, 0.1 mL) as substrate, in Tris–HCl buffer pH = 9.0. The test samples dissolved in methanol (0.1 mL) were added to the mixture (total volume of 3 mL) and the reaction was monitored for 5 min at 28 °C recording absorbance at 234 nm. Each concentration was evaluated twice and the results were expressed as IC_50_ (µM) after incubation for 5 min. Blank samples were prepared without the lipoxygenase enzyme, along with a control that lacked the extract sample [103,104,105]. Ferulic acid and naproxen, both standard compounds with known lipoxygenase-inhibitory activity, were used as reference substances. All experiments were performed in triplicate to ensure reproducibility and reliability of the results.

#### 3.6.6. Cyclooxygenase Inhibition

In vitro COX inhibition activity was performed with a COX-1,2 screening assay that directly measures PGF_2a_ formed by SnCl_2_ reduction of COX-derived PGH_2_ (Cayman Chemical, Item No. 560131). The substrate used for COX-1,2 was arachidonic acid (10 μM final concentration). All extracts were tested at 100 and 50 μg/mL. The prostanoid product is quantified via an Enzyme Immunoassay (EIA) using a broadly specific antibody that binds to all major prostaglandin derivatives. The method is based on the competition between the PG product and a PG-acetylcholinesterase conjugate for a limited amount of PG antiserum. Cyclooxygenase enzymes used were ovine, for COX-1, and human recombinant, for COX-2. Blank samples were prepared without cyclooxygenase enzymes, along with a control that lacked the extract sample [106]. Indomethacin, a compound with known cyclooxygenase 1- and 2-inhibitory activity, was used as a reference substance at a concentration of 7.16 μg/mL. All experiments were performed in triplicate to ensure reproducibility and reliability of the results.

### 3.7. In Vivo Biological Assay

#### 3.7.1. Animals

Animal care was performed according to the guidelines established by the European Council Directive 2010/63/EU. This experimental procedure was approved by the National Peripheral Veterinary Authority Animal Ethics Committee after the affirmative opinion by the Animal Protocols Evaluation Committee (Ethics approval #342725/14-8-2024). Female C57BL/6 mice (3–7 months old) were used in this study. All mice originated from the breeding stock of the Small Animal Laboratory of the Section of Pharmaceutical Technology, Department of Pharmacy (EL 25 BIO 06). The animal’s room temperature and humidity were maintained at 24 ± 1 °C and 45%, respectively. The room was illuminated by yellow-fluorescent tubes in a 12 h cycle of light and dark (switched on at 8:00 and off at 20:00); these lamps do not emit any measurable UV radiation. The mice had unrestricted continuous access to standard solid pellets (Nuevo SA-Farma-Efyra Industrial & Commercial SA, Greece) and fresh water.

#### 3.7.2. Carrageenan-Induced Paw Edema Reduction Assay

For the in vivo anti-inflammatory activity, C57BL/6 female mice (3–7 months old, 20–34 g body weight) were randomly assigned to a group (6 animals/group, for a total of 36 animals) and were injected with 0.025 mL carrageenan (Merck KGaA, Darmstadt, Germany) (2% *w*/*v* solution in saline) i.d. into the right hind paw, with the left paw serving as the control. The test extracts *C. creticus*, *C. coggygria*, *H. empetrifolium*, *T. chamaedrys* and *O. dictamnus*, dissolved in saline (0.03 mg/g body weight, administered in a volume of 0.01 mL/g body weight) [105,107] with a few drops of Tween-80, were administered i.p. right after the carrageenan injection. After 3.5 h, mice were sacrificed, their hind legs were removed, and their weights were measured in a precision analytical balance. The produced edema was estimated as the difference in weight (g) between the challenged right paw and the left paw. Results were expressed as the percentage of reduction in paw edema (mean from six animals per extract) [90,105].

### 3.8. Statistical Analysis

Results are expressed as mean ± standard deviation (SD). Statistical analyses were conducted using GraphPad Prism software, version 8.0.1 (GraphPad Software, Inc., San Diego, CA, USA). In the in vivo experiments, which involved more than two experimental groups, data were initially evaluated for normality. After verification that the assumptions required for parametric analysis were fulfilled, a parametric one-way analysis of variance (ANOVA) was applied to assess statistically significant differences between the experimental groups and the control group. A *p*-value of ≤0.05 was considered indicative of statistical significance.

## 4. Conclusions

In the present study, seventeen different plant species belonging to the Balkan flora were evaluated for their antioxidant, anti-inflammatory and skin-related enzyme inhibitory properties, using both in vitro and in vivo methods. The preliminary results presented hereby demonstrate the broad antioxidant and anti-inflammatory potential of several Balkan medicinal plant extracts, supporting their traditional use in the treatment of related skin disorders. The highest antioxidant capacity in vitro was presented by the extracts of *C. coggygria*, *C. creticus*, *H. empetrifolium*, *S. thymbra*, *S. sediforme* and *T. chamaedrys* under the tested conditions, while the extracts of *C. creticus* and *S. sediforme* demonstrated potential anti-inflammatory activity in the carrageenan-induced paw edema model, as well as in the in vitro inhibition assays of COX-1, COX-2 and LOX. Their high radical scavenging activity can be explained by the presence of a large amount of phenolic and flavonoid compounds. Thus, the above extracts could be used as a promising source of preventive/therapeutic agents with a beneficial effect on the pathogenesis/development of several inflammatory and oxidative stress-related disorders. To advance these findings, future research should include comprehensive chemical characterization, in order to identify the key bioactive components, as well as studies such as enzyme inhibition kinetics, receptor-binding assays, and cellular pathway analyses to elucidate the molecular mode of action. Additionally, in vivo pharmacokinetic studies would assess the absorption, distribution, metabolism, and excretion (ADME) of active compounds, alongside efficacy testing in relevant disease models. These combined approaches will provide a clearer understanding of the therapeutic potential, mechanism of action, and safety profile of the most bioactive extracts for potential use in cosmetic and pharmaceutical products.

## Figures and Tables

**Table 1:** The percentage yields of the produced plant extracts.

Code Name	Yield (%)	Code Name	Yield (%)	Code Name	Yield (%)
*C. salonitana* D	3.0	*C. salonitana* M	6.0	*C. salonitana* WM	7.5
*C. creticus* D	2.4	*C. creticus* M	13.0	*C. creticus* WM	7.7
*C. coggygria* D	2.4	*C. coggygria* M	18.7	*C. coggygria* WM	15.7
*D. graveolens* D	5.3	*D. graveolens* Μ	5.3	*D. graveolens* WM	4.7
*E. graecus* a.p. ^1^ D	1.0	*E. graecus* a.p. ^1^ M	7.5	*E. graecus* a.p. ^1^ WM	7.5
*E. graecus* roots D	3.6	*E. graecus* roots M	7.2	*E. graecus* roots WM	6.5
*H. empetrifolium* D	8.7	*H. empetrifolium* M	11.6	*H. empetrifolium* WM	10.2
*J. regia* D	2.5	*J. regia* M	22.1	*J. regia* WM	17.2
*J. oxycedrus* D	4.4	*J. oxycedrus* M	11.5	*J. oxycedrus* WM	7.0
*O. dictamnus* D	6.4	*O. dictamnus* M	5.0	*O. dictamnus* WM	12.1
*P. nigra* D	5.5	*P. nigra* M	10.5	*P. nigra* WM	4.0
*P. bituminosa* D	2.2	*P. bituminosa* M	10.3	*P. bituminosa* WM	11.8
*S. fruticosa* D	6.9	*S. fruticosa* M	6.1	*S. fruticosa* WM	14.9
*S. nigra* D	4.0	*S. nigra* M	11.2	*S. nigra* WM	16.2
*S. thymbra* D	2.2	*S. thymbra* M	6.2	*S. thymbra* WM	10.6
*S. sediforme* D	4.0	*S. sediforme* M	3.0	*S. sediforme* WM	3.8
*T. chamaedrys* D	3.7	*T. chamaedrys* M	5.5	*T. chamaedrys* WM	20.3
*T. capitata* D	1.9	*T. capitata* M	5.3	*T. capitata* WM	6.3

^1^ a.p. = aerial parts.

**Table 2:** Total phenolic content (TPC) as mg GAE ^2^/g of dw of plant extracts (mean ± SD ^1^).

Code Name	TPC (Mean ± SD ^1^)	Code Name	TPC (Mean ± SD ^1^)	Code Name	TPC (Mean ± SD ^1^)
*C. salonitana* D	29.9 (±2.0)	*C. salonitana* M	49.6 (±1.1)	*C. salonitana* WM	38.0 (±0.3)
*C. creticus* D	12.0 (±0.3)	*C. creticus* M	79.8 (±2.4)	*C. creticus* WM	169.8 (±2.2)
*C. coggygria* D	25.3 (±1.4)	*C. coggygria* M	167.8 (±10.7)	*C. coggygria* WM	123.2 (±5.2)
*D. graveolens* D	4.5 (±0.2)	*D. graveolens* Μ	30.6 (±0.4)	*D. graveolens* WM	82.3 (±4.2)
*E. graecus* a.p. ^3^ D	0.7 (±0.5)	*E. graecus* a.p. ^3^ M	30.8 (±1.3)	*E. graecus* a.p. ^3^ WM	41.1 (±0.9)
*E. graecus* roots D	14.6 (±0.1)	*E. graecus* roots M	4.9 (±0.2)	*E. graecus* roots WM	10.7 (±0.2)
*H. empetrifolium* D	44.8 (±0.6)	*H. empetrifolium* M	97.6 (±1.0)	*H. empetrifolium* WM	109.6 (±3.0)
*J. regia* D	21.6 (±0.6)	*J. regia* M	66.6 (±3.4)	*J. regia* WM	99.1 (±0.3)
*J. oxycedrus* D	16.8 (±0.7)	*J. oxycedrus* M	92.6 (±3.4)	*J. oxycedrus* WM	104.8 (±4.7)
*O. dictamnus* D	33.6 (±1.3)	*O. dictamnus* M	74.3 (±1.4)	*O. dictamnus* WM	102.2 (±4.4)
*P. nigra* D	14.1 (±0.9)	*P. nigra* M	60.8 (±0.8)	*P. nigra* WM	57.0 (±1.7)
*P. bituminosa* D	40.6 (±0.2)	*P. bituminosa* M	25.6 (±1.1)	*P. bituminosa* WM	29.7 (±0.1)
*S. fruticosa* D	35.3 (±0.3)	*S. fruticosa* M	108.7 (±0.9)	*S. fruticosa* WM	99.8 (±2.5)
*S. nigra* D	2.7 (±0.1)	*S. nigra* M	52.2 (±1.2)	*S. nigra* WM	47.1 (±2.4)
*S. thymbra* D	59.2 (±1.5)	*S. thymbra* M	104.3 (±1.4)	*S. thymbra* WM	121.1 (±3.7)
*S. sediforme* D	13.5 (±0.8)	*S. sediforme* M	106.1 (±0.7)	*S. sediforme* WM	95.1 (±0.2)
*T. chamaedrys* D	35.8 (±2.0)	*T. chamaedrys* M	111.8 (±0.1)	*T. chamaedrys* WM	119.1 (±6.3)
*T. capitata* D	24.4 (±2.1)	*T. capitata* M	99.8 (±0.8)	*T. capitata* WM	78.9 (±0.5)

^1^ SD = Standard deviation, ^2^ GAE = Gallic acid equivalents, ^3^ a.p. = aerial parts.

**Table 3:** Total flavonoid content (TFC) as mg QUE ^2^/g dry plant extract (± SD ^1^).

Code Name	TFC (Mean ± SD ^1^)	Code Name	TFC (Mean ± SD ^1^)	Code Name	TFC (Mean ± SD ^1^)
*C. salonitana* D	8.3 (±1.9)	*C. salonitana* M	10.5 (±1.2)	*C. salonitana* WM	13.7 (±0.8)
*C. creticus* D	3.8 (±1.8)	*C. creticus* M	18.5 (±1.8)	*C. creticus* WM	24.3 (±10.5)
*C. coggygria* D	4.8 (±1.1)	*C. coggygria* M	18.9 (±2.3)	*C. coggygria* WM	25.9 (±0.8)
*D. graveolens* D	3.5 (±0.9)	*D. graveolens* Μ	21.3 (±3.0)	*D. graveolens* WM	41.1 (±5.5)
*E. graecus* a.p. ^4^ D	0.5 (±1.2)	*E. graecus* a.p. ^4^ M	19.4 (±4.8)	*E. graecus* a.p. ^4^ WM	15.6 (±2.0)
*E. graecus* roots D	ND ^3^	*E. graecus* roots M	1.9 (±1.2)	*E. graecus* roots WM	1.5 (±2.4)
*H. empetrifolium* D	ND ^3^	*H. empetrifolium* M	30.4 (±4.5)	*H. empetrifolium* WM	24.7 (±5.7)
*J. regia* D	8.5 (±1.3)	*J. regia* M	58.3 (±1.3)	*J. regia* WM	54.8 (±2.3)
*J. oxycedrus* D	ND ^3^	*J. oxycedrus* M	ND ^3^	*J. oxycedrus* WM	ND ^3^
*O. dictamnus* D	ND ^3^	*O. dictamnus* M	15.9 (±9.3)	*O. dictamnus* WM	38.9 (±4.0)
*P. nigra* D	1.6 (±0.5)	*P. nigra* M	3.6 (±0.5)	*P. nigra* WM	10.3 (±0.8)
*P. bituminosa* D	ND ^3^	*P. bituminosa* M	12.3 (±0.3)	*P. bituminosa* WM	15.4 (±0.4)
*S. fruticosa* D	8.1 (±1.3)	*S. fruticosa* M	40.1 (±2.3)	*S. fruticosa* WM	45.5 (±2.2)
*S. nigra* D	2.2 (±2.4)	*S. nigra* M	45.8 (±8.3)	*S. nigra* WM	42.5 (±8.3)
*S. thymbra* D	8.5 (±1.1)	*S. thymbra* M	22.6 (±1.0)	*S. thymbra* WM	23.4 (±0.9)
*S. sediforme* D	2.5 (±0.4)	*S. sediforme* M	22.5 (±1.5)	*S. sediforme* WM	6.1 (±0.4)
*T. chamaedrys* D	14.7 (±2.6)	*T. chamaedrys* M	42.9 (±0.4)	*T. chamaedrys* WM	33.8 (±1.3)
*T. capitata* D	6.6 (±0.1)	*T. capitata* M	33.5 (±2.3)	*T. capitata* WM	14.6 (±1.5)

^1^ SD = Standard deviation, ^2^ QUE = Quercetin equivalents, ^3^ ND = Not Detected, ^4^ a.p. = aerial parts.

**Table 4:** Free radical scavenging activity of plant extracts expressed as % DPPH inhibition at 200, 100 and 50 μg/mL (mean ± SD ^1^).

Code Name	% Inhibition at 200 μg/mL (Mean ± SD ^1^)	Code Name	% Inhibition at 200 μg/mL (Mean ± SD ^1^)	% Inhibition at 100 μg/mL (Mean ± SD ^1^)	% Inhibition 50 μg/mL (Mean ± SD ^1^)	Code Name	% Inhibition at 200 μg/mL (Mean ± SD ^1^)	% Inhibition at 100 (Mean ± SD ^1^)	% Inhibition 50 μg/mL (Mean ± SD ^1^)
*C. salonitana* D	11.7 (±1.5)	*C. salonitana* M	35.7 (±1.4)	-	-	*C. salonitana* WM	17.9 (±0.1)	-	-
*C. creticus* D	15.1 (±0.8)	*C. creticus* M	93.7 (±0.1)			*C. creticus* WM	94.3 (±0.1)		
*C. coggygria* D	33.6 (±6.6)	*C. coggygria* M	96.4 (±0.1)	95.1 (±0.5)	67.7 (±0.4)	*C. coggygria* WM	95.9 (±0.1)	95.8 (±0.2)	52.8 (±2.6)
*D. graveolens* D	3.5 (±1.1)	*D. graveolens* Μ	52.7 (±6.1)			*D. graveolens* WM	74.0 (±2.2)		
*E. graecus* a.p. ^3^ D	7.0 (±1.0)	*E. graecus* a.p. ^3^ M	47.4 (±2.1)	21.7 (±1.4)	-	*E. graecus* a.p. ^3^ WM	50.9 (±2.8)	26.4 (±1.4)	-
*E. graecus* roots D	5.8 (±0.8)	*E. graecus* roots M	12.5 (±0.7)	6.2 (±0.5)	-	*E. graecus* roots WM	19.4 (±0.4)	8.7 (±0.7)	-
*H. empetrifolium* D	46.8 (±2.1)	*H. empetrifolium* M	86.6 (±2.8)	NT ^4^	NT ^4^	*H. empetrifolium* M	93.0 (±0.2)	NT ^4^	NT ^4^
*J. regia* D	18.3 (±3.3)	*J. regia* M	86.0 (±3.5)	59.8 (±0.1)	33.1 (±0.4)	*J. regia* WM	88.5 (±1.9)	74.6 (±6.8)	36.5 (±2.0)
*J. oxycedrus* D	12.2 (±1.1)	*J. oxycedrus* M	93.5 (±0.3)	90.3 (±1.4)	59.1 (±3.7)	*J. oxycedrus* WM	92.7 (±1.4)	79.6 (±2.9)	47.1 (±3.3)
*O. dictamnus* D	27.6 (±3.4)	*O. dictamnus* M	90.0 (±0.4)	NT ^4^	NT ^4^	*O. dictamnus* WM	89.7 (±0.5)	NT ^4^	NT ^4^
*P. nigra* D	6.6 (±0.8)	*P. nigra* M	86.5 (±1.3)	61.1 (±2.1)	NT ^4^	*P. nigra* WM	85.8 (±0.9)	52.7 (±0.5)	-
*P. bituminosa* D	22.4 (±0.1)	*P. bituminosa* M	16.6 (±0.3)	-	-	*P. bituminosa* WM	9.4 (±1.1)	NT ^4^	NT ^4^
*S. fruticosa* D	63.3 (±1.9)	*S. fruticosa* M	90.3 (±0.9)	75.9 (±1.1)	39.7 (±1.5)	*S. fruticosa* WM	92.8 (±0.7)	69.0 (±1.1)	-
*S. nigra* D	ND ^2^	*S. nigra* M	85.5 (±2.7)	NT ^4^	NT ^4^	*S. nigra* WM	72.3 (±5.0)	NT ^4^	NT ^4^
*S. thymbra* D	38.1 (±0.9)	*S. thymbra* M	91.0 (±0.8)	73.8 (±3.1)	38.3 (±0.9)	*S. thymbra* WM	90.4 (±0.5)	80.6 (±0.3)	42.4 (±0.8)
*S. sediforme* D	11.3 (±0.8)	*S. sediforme* M	94.8 (±0.3)	83.8 (±1.2)	53.8 (±1.2)	*S. sediforme* WM	94.8 (±0.3)	77.5 (±1.0)	51.1 (±0.4)
*T. chamaedrys* D	41.3 (±1.8)	*T. chamaedrys* M	92.4 (±0.3)	71.3 (±0.7)	34.8 (±0.5)	*T. chamaedrys* WM	91.0 (±0.2)	85.4 (±0.7)	44.0 (±7.8)
*T. capitata* D	16.2 (±1.0)	*T. capitata* M	90.6 (±1.5)	62.5 (±1.3)	31.2 (±1.1)	*T. capitata* WM	88.6 (±1.1)	53.9 (±0.7)	30.2 (±0.8)

^1^ SD = Standard deviation, ^2^ ND = Not detected, ^3^ a.p. = Aerial parts, ^4^ NT: Not tested.

**Table 5:** % Tyrosinase and collagenase inhibitory activity of the plant extracts (mean± SD ^1^).

Code Name	Tyrosinase Inhibition Assay	Collagenase Inhibition Assay	Code Name	Tyrosinase Inhibition Assay	Collagenase Inhibition Assay
	%Inhibition at 300 μg/mL) (Mean ± SD ^1^)	%Inhibition at 100 μg/mL (Mean ± SD ^1^)		%Inhibition at 300 μg/mL (Mean ± SD ^1^)	%Inhibition at 100 μg/mL (Mean ± SD ^1^)
*C. salonitana* M	80.7 (±1.7)	30.0 (±0.3)	*C. salonitana* WM	12.5 (±0.3)	21.4 (±2.8)
*C. creticus* M	94.2 (±1.1)	96.1 (±1.2)	*C. creticus* WM	93.3 (±0.4)	94.7 (±3.0)
*C. coggygria* M	83.1 (±0.3)	94.8 (±0.5)	*C. coggygria* WM	94.2 (±0.6)	86.8 (±1.7)
*D. graveolens* Μ	45.7 (±0.4)	46.1 (±0.5)	*D. graveolens* WM	48.9 (±0.4)	64.7 (±5.5)
*E. graecus* a.p. ^3^ M	44.1 (±0.6)	26.7 (±1.0)	*E. graecus* a.p. ^3^ WM	48.0 (±1.1)	9.8 (±4.2)
*E. graecus* roots M	14.6 (±3.8)	8.0 (±4.2)	*E. graecus* roots WM	12.0 (±1.4)	ND ^2^
*H. empetrifolium* M	37.6 (±0.6)	79.4 (±0.3)	*H. empetrifolium* WM	20.5 (±7.2)	72.9 (±2.3)
*J. regia* M	ND ^2^	11.6 (±0.4)	*J. regia* WM	ND ^2^	21.7 (±0.1)
*J. oxycedrus* M	46.2 (±2.5)	37.5 (±0.2)	*J. oxycedrus* WM	48.5 (±1.9)	23.5 (±0.6)
*O. dictamnus* M	44.2 (±0.1)	71.0 (±2.8)	*O. dictamnus* WM	42.6 (±0.1)	98.8 (±1.2)
*P. nigra* M	92.1 (±0.6)	69.0 (±2.1)	*P. nigra* WM	61.7 (±1.9)	52.9 (±1.2)
*P. bituminosa* M	9.9 (±0.3)	18.2 (±0.2)	*P. bituminosa* WM	13.6 (±3.0)	39.7 (±3.8)
*S. fruticosa* M	29.4 (±2.2)	59.3 (±0.6)	*S. fruticosa* WM	33.0 (±0.9)	42.7 (±2.3)
*S. nigra* M	44.9 (±1.2)	33.1 (±2.0)	*S. nigra* WM	37.1 (±2.0)	26.7 (±1.5)
*S. thymbra* M	37.5 (±0.9)	54.0 (±1.1)	*S. thymbra* WM	39.8 (±1.6)	39.6 (±0.3)
*S. sediforme* M	81.4 (±2.4)	83.6 (±1.4)	*S. sediforme* WM	78.1 (±0.9)	89.6 (±1.4)
*T. chamaedrys* M	37.6 (±1.6)	51.0 (±3.2)	*T. chamaedrys* WM	26.4 (±0.7)	62.9 (±0.6)
*T. capitata* M	31.9 (±0.4)	55.8 (±0.4)	*T. capitata* WM	56.7 (±1.1)	34.5 (±0.4)

^1^ SD = Standard deviation, ^2^ ND = Not detected, ^3^ a.p. = Aerial parts.

**Table 6:** Chelating capacity of Fe^2+^ of the medicinal plants at 200 μg/mL and their IC_50_ values (mean ± SD ^1^).

Code Name	% Free Fe^+2^ at 200 μg/mL (Mean ± SD ^1^)	IC_50_ Value (μg/mL) (Mean ± SD ^1^)
*C. salonitana* M	76.8 (±1.8)	300.0 (±9.3)
*C. creticus* M	30.0 (±2.3)	103.0 (±2.2)
*C. coggygria* M	10.8 (±1.8)	37.0 (±1.1)
*D. graveolens* WM	18.8 (±2.8)	25.0 (±1.5)
*E. graecus* a.p. ^2^ M	82.4 (±6.2)	ND ^3^
*E. graecus* roots M	89.2 (±5.6)	ND ^3^
*H. empetrifolium* WM	1.7 (±0.5)	5.0 (±1.7)
*J. regia* WM	49.4 (±5.1)	201.3 (±1.1)
*J. oxycedrus* WM	21.3 (±1.7)	255.8 (±2.9)
*O. dictamnus* WM	7.9 (±2.9)	70.6 (±1.4)
*P. nigra* WM	19.9 (±1.7)	118.5 (±1.0)
*P. bituminosa* M	62.5 (±4.5)	ND ^3^
*P. bituminosa* WM	70.5 (±5.8)	ND ^3^
*S. fruticosa* WM	14.0 (±3.8)	121.1 (±1.0)
*S. nigra* WM	34.7 (±1.7)	133.6 (±1.6)
*S. thymbra* WM	5.1 (±2.0)	86.1 (±1.1)
*S. sediforme* M	15.0 (±1.9)	26.0 (±2.2)
*T. chamaedrys* WM	12.3 (±2.1)	99.8 (±2.1)
*T. capitata* M	31.6 (±2.0)	150.5 (±8.7)
EDTA	1.0 (±0.1)	ND ^3^

^1^ SD = Standard deviation, ^2^ a.p. = Aerial parts, ^3^ ND: Not determined.

**Table 7:** Inhibitory activities of the plant extracts against soybean lipoxygenase-3 as IC_50_ values (or % inhibition at 200 μg/mL), after 5 min of incubation.

Code Name	% Inhibition at 200 μg/mL (Mean ± SD ^1^)	IC_50_ Value (μg/mL) (Mean ± SD ^1^)
*C. salonitana* M	ND ^2^	-
*C. creticus* M	95.3 (±0.1)	104.3 (±1.0)
*C. coggygria* M	99.4 (±0.3)	<100
*D. graveolens* WM	40.0 (±3.3)	-
*E. graecus* a.p. ^3^ M	21.2 (±0.7)	-
*E. graecus* roots M	ND ^2^	-
*H. empetrifolium* WM	88.8 (±0.9)	103.0 (±1.0)
*J. regia* WM	60.1 (±2.3)	192.6 (±1.9)
*J. oxycedrus* WM	67.3 (±1.0)	147.3 (±1.1)
*O. dictamnus* WM	56.2 (±1.9)	143.3 (±1.2)
*P. nigra* WM	77.7 (±0.7)	132.0 (±1.6)
*P. bituminosa* M	53.4 (±0.6)	200.0 (±2.2)
*P. bituminosa* WM	32.3 (±0.4)	-
*S. fruticosa* WM	47.7 (±0.4)	210.7 (±2.5)
*S. nigra* WM	12.9 (±0.2)	-
*S. thymbra* WM	72.4 (±1.1)	173.7 (±1.0)
*S. sediforme* M	94.3 (±2.7)	39.4 (±1.1)
*T. chamaedrys* WM	48.1 (±0.7)	201.3 (±1.0)
*T. capitata* M	84.6 (±0.5)	28.0 (±1.2)
*ferulic acid*	90 (±0.3)	25.6(±0.5)
*naproxen*	97 (±0.5)	5.76 (±0.8)

^1^ SD = Standard deviation, ^2^ ND = Not detected, ^3^ a.p. = Aerial parts.

**Table 8:** Botanical name of the examined plant species, along with their family, origin, plant part, harvest season, code name and extraction solvent.

Botanical Name	Family	Origin	Lat/Lon	Plant Part	Harvest Season	Code Name	Extraction Solvent
*Centaurea salonitana* Vis.	Asteraceae	Molos, Phthiotis, Greece	38.8087 N, 22.6450 E|EPSG:4326	Aerial parts	August 2020	ETHGR0307D	DCM ^1^
						ETHGR0307M	Methanol
						ETHGR0307WM	Water-Methanol (1:1)
*Cistus creticus* L. ssp. *creticus*	Cistaceae	Mount Hymettus, Attica, Greece	37.9630 N, 23.8167 E|EPSG:4326	Aerial parts	May 2020	ETHGR0111D	DCM^1^
						ETHGR0111M	Methanol
						ETHGR0111WM	Water-Methanol (1:1)
*Cotinus coggygria* Scop.	Anacardiaceae	Devojački Bunar, Serbia	44.98988 N, 20.94709 E|EPSG:4326	Herba	July 2021	ETHSR0021D	DCM ^1^
						ETHSR0021M	Methanol
						ETHSR0021WM	Water-Methanol (1:1)
*Dittrichia graveolens* (L.) Greuter	Compositae	Markopoulo mesogaias, Attica, Greece	37.883356 N, 23.933304 E|EPSG:4326	Wood plant	October 2020	ETHGR0398D	DCM ^1^
						ETHGR0398M	Methanol
						ETHGR0398WM	Water-Methanol (1:1)
*Echinops graecus* Mill.	Asteraceae	Nea Peramos, Attica, Greece	38.000 N, 23.417 E|EPSG:4326	Aerial parts	July 2020	ETHGR0300D	DCM ^1^
						ETHGR0300M	Methanol
						ETHGR0300WM	Water-Methanol (1:1)
*Echinops graecus* Mill.	Asteraceae	Nea Peramos, Attica, Greece	38.000 N, 23.417 E|EPSG:4326	Roots	July 2020	ETHGR0302D	DCM ^1^
						ETHGR0302M	Methanol
						ETHGR0302WM	Water-Methanol (1:1)
*Hypericum empetrifolium* Willd.	Hypericaceae	Panepistimiopolis Zografou, Athens, Greece	37.97151 N, 23.76268 E|EPSG:4326	Aerial parts	May 2020	ETHGR0119D	DCM ^1^
						ETHGR0119M	Methanol
						ETHGR0119WM	Water-Methanol (1:1)
*Juglans regia* L.	Juglandaceae	Prespes, West Macedonia, Greece	40.840557 N, 21.161493 E |EPSG:4326	Leaves-Branches	May 2021	ETHGR0355D	DCM ^1^
						ETHGR0355M	Methanol
						ETHGR0355WM	Water-Methanol (1:1)
*Juniperus oxycedrus* L. ssp. *deltoides*	Cupressaceae	Leonidio, Peloponnese, Greece	37.1673 N, 22.8593 E|EPSG:4326	Aerial parts	June 2020	ETHGR0221D	DCM ^1^
						ETHGR0221M	Methanol
						ETHGR0221WM	Water-Methanol (1:1)
*Origanum dictamnus* L.	Lamiaceae	Crete, Greece	35.09280 N, 25.38321 E|EPSG:4326	Aerial parts	August 2021	ETHGR0403D	DCM ^1^
						ETHGR0403M	Methanol
						ETHGR0403WM	Water-Methanol (1:1)
*Pinus nigra* J.F.Arnold	Pinaceae	Aiani Kozanis, West Macedonia, Greece	40.1646 N, 21.8197 E|EPSG:4326	Aerial parts	June 2020	ETHGR0184D	DCM ^1^
						ETHGR0184M	Methanol
						ETHGR0184WM	Water-Methanol (1:1)
*Psoralea bituminosa* L.	Leguminosae	Panepistimiopolis Zografou, Athens, Greece	37.97151 N, 23.76268 E|EPSG:4326	Roots	April 2020	ETHGR0075D	DCM ^1^
						ETHGR0075M	Methanol
						ETHGR0075WM	Water-Methanol (1:1)
*Salvia fruticosa* Mill.	Lamiaceae	Mount Hymettus, Attica, Greece	37.9630 N, 23.8167 E|EPSG:4326	Leaves- Aerial parts- Stems	May 2020	ETHGR0136D	DCM ^1^
						ETHGR0136M	Methanol
						ETHGR0136WM	Water-Methanol (1:1)
*Sambucus nigra* L.	Caprifoliaceae	Prespes, West Macedonia, Greece	40.76374 N, 21.14142 E |EPSG:4326	Influoresence	July 2021	ETHGR0385D	DCM ^1^
						ETHGR0385M	Methanol
						ETHGR0385WM	Water-Methanol (1:1)
*Satureja thymbra* L.	Lamiaceae	Mount Hymettus, Attica, Greece	37.9630 N, 23.8167 E|EPSG:4326	Aerial parts	May 2020	ETHGR0137D	DCM ^1^
						ETHGR0137M	Methanol
						ETHGR0137WM	Water-Methanol (1:1)
*Sedum sediforme* (Jacq.) Pau	Crassulaceae	Mount Hymettus, Attica, Greece	37.9630 N, 23.8167 E|EPSG:4326	Aerial parts	May 2020	ETHGR0138D	DCM ^1^
						ETHGR0138M	Methanol
						ETHGR0138WM	Water-Methanol (1:1)
*Teucrium chamaedrys* L.	Lamiaceae	Devojački Bunar, Serbia	44.98988 N, 20.94709 E|EPSG:4326	Aerial parts	May 2021	ETHSR0063D	DCM ^1^
						ETHSR0063M	Methanol
						ETHSR0063WM	Water-Methanol (1:1)
*Thymbra capitata* L. Cav.	Lamiaceae	Leonidio, Peloponnese, Greece	37.9630 N, 23.8167 E|EPSG:4326	Herba	June 2020	ETHGR0248D	DCM ^1^
						ETHGR0248M	Methanol
						ETHGR0248WM	Water-Methanol (1:1)

^1^ DCM = Dichloromethane.

## Data Availability

The original contributions presented in this study are included in the article. Further inquiries can be directed to the corresponding author.

## Supplementary Materials

The following supporting information can be downloaded at https://www.mdpi.com/article/10.3390/molecules31091524/s1, Figure S1: Chromatograms of the dichloromethane, methanolic and hydroalcoholic extracts in the visible, 254 and 366 nm wavelengths, sprayed with vanillin sulfate, heated in a heating plate and re-photographed in the visible and at 366 nm wavelengths. In the case of HPTLC-DPPH bioautographic evaluation, the plates were photographed, after immersion in a DPPH methanolic solution (0.05% *w*/*v*), both in visible light and at a wavelength of 366 nm, respectively.; Figure S2: Gallic acid calibration curve. From the reference curve, the linear segment was selected and based on the equation y = 0.0745x + 0.0896, the gallic acid equivalents (GAE) for each sample were calculated (mg GAE per 100 mg dry sample). Samples that exhibited activity within the linear range of the reference curve were accepted, while those showing absorbance outside this range were retested at lower concentrations; Figure S3: Querqetin calibration curve. From the black segment of the curve (with limits 0.500 < A < 3.300), the corresponding quercetin concentration was calculated and then the equivalent concentration in μg/ml. Samples that exhibited activity within the linear range of the reference curve were accepted, while those showing absorbance outside this range were retested at lower concentrations.
