# Supplementary material for: Exploring the Antioxidant, Anti-Inflammatory and Skin-Enzyme Inhibitory Activities of Balkan Ethnomedicinal Herbs Through In Vitro and In Vivo Screening
Source: Molecules. 2026 May 4;31(9):1524. doi: 10.3390/molecules31091524 (PMC13165090; doi:10.3390/molecules31091524)
Supplement: Supplementary file 1 [file molecules-31-01524-s001.zip › molecules-4132909-supplementary.pdf]

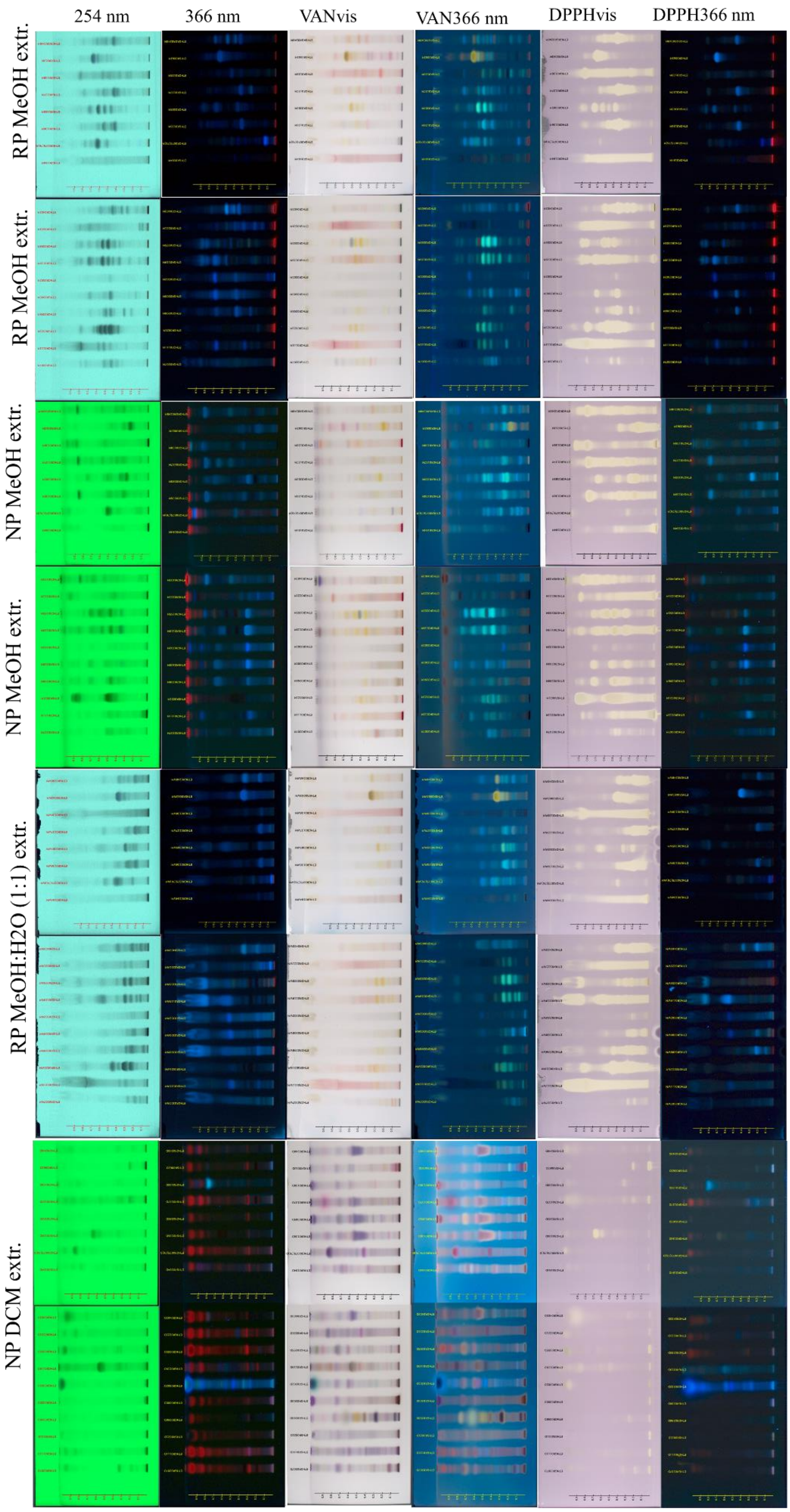


**Figure S1.** Chromatograms of the dichloromethane, methanolic and hydroalcoholic extracts in the visible, 254 and 366 nm wavelengths, sprayed with vanillin sulfate, heated in a heating plate and re-photographed in the visible and at 366 nm wavelengths. In the case of HPTLC-DPPH bioautographic evaluation, the plates were photographed, after immersion in a DPPH methanolic solution (0.05% w/v), both in visible light and at a wavelength of 366 nm, respectively.

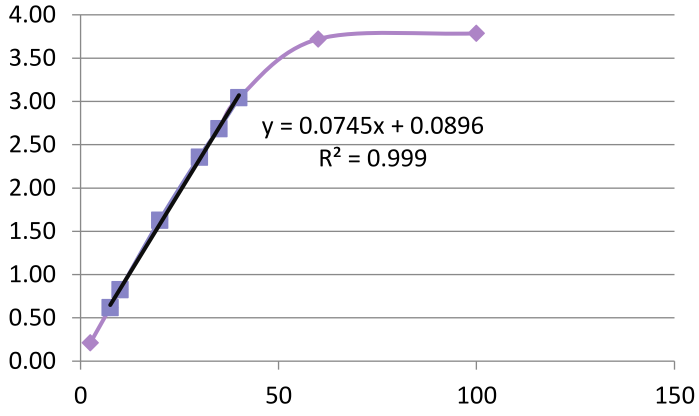


**Figure S2.** Gallic acid calibration curve. From the reference curve, the linear segment was selected and based on the equation y = 0.0745x + 0.0896, the gallic acid equivalents (GAE) for each sample were calculated (mg GAE per 100 mg dry sample). Samples that exhibited activity within the linear range of the reference curve were accepted, while those showing absorbance outside this range were retested at lower concentrations.

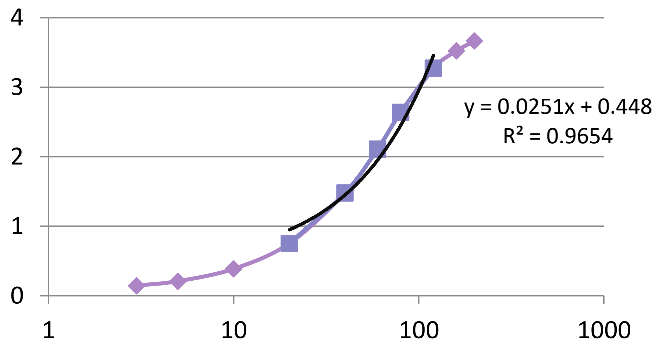


**Figure S3.** Querqetin calibration curve. From the black segment of the curve (with limits 0.500 < A < 3.300), the corresponding quercetin concentration was calculated and then the equivalent concentration in μg/ml. Samples that exhibited activity within the linear range of the reference curve were accepted, while those showing absorbance outside this range were retested at lower concentrations [92].
